# The Heat-Shock Metabolite Streptolactam D, Produced by High-Temperature Culture of *Streptomyces* sp. JA74, Promotes Thermotolerance via Self-Membrane Stabilization

**DOI:** 10.1021/jacs.5c03026

**Published:** 2025-04-28

**Authors:** Shun Saito, Yurika Okumura, Sosuke Kataoka, Keisuke Fukaya, Daisuke Urabe, Midori A. Arai

**Affiliations:** † Department of Biosciences and Informatics, Keio University, 3-14-1 Hiyoshi, Kohoku-ku, Yokohama 223-8522, Japan; ‡ Biotechnology Research Center and Department of Biotechnology, Toyama Prefectural University, 5180 Kurokawa, Imizu, Toyama 939-0398, Japan

## Abstract

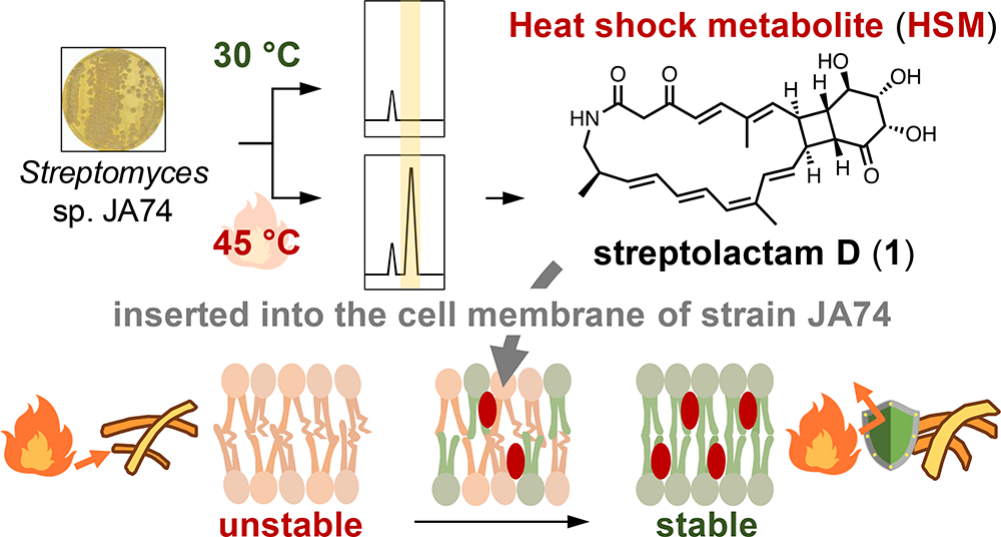

A new streptolactam derivative featuring a 4-membered ring fused to a 6-membered ring in the macrolactam structure, streptolactam D (**1**), was isolated from the culture extract of thermotolerant *Streptomyces* sp. JA74. Production of compound **1** increased with high-temperature cultivation, and this type of compound was previously designated as a “heat-shock metabolite (HSM)” by our research group. The structure of **1** was determined by NMR and MS spectroscopic analyses, calculation of NMR and ECD spectra, and analysis of the biosynthetic gene cluster. Surprisingly, **1** promoted the growth of strain JA74 under high-temperature conditions and increased the growth limit temperature. Analysis of the mode of action using ultracentrifugation and scanning electron microscopy suggested that **1** localizes in the cell membrane and causes the cell to assume a short and thick morphology. Furthermore, **1** is predicted to maintain cell membrane fluidity and impart thermotolerance to strain JA74, similar to cholesterol and saturated fatty acids. These data suggest that **1** is produced to promote the survival of strain JA74 under high-temperature conditions.

## Introduction

Secondary metabolites (SMs) produced by actinomycetes have demonstrated usefulness in a variety of fields and secured a firm position as a source of small molecular compounds.^1,2^ However, obtaining novel small molecular compounds from actinomycetes has become increasingly difficult. One of the reasons for this difficulty is that many of the biosynthetic genes for SMs produced by actinomycetes are in a silent state under normal culture conditions. Therefore, intensive research is focused on the development of new methods to search for compounds that activate such silent biosynthetic genes.^3,4^ However, details regarding the physiological functionality of SMs (i.e., why actinomycetes activate silent genes and produce these compounds) remain largely unexplained. Our group previously discovered a phenomenon in which actinomycetes produce metabolites when cultured at high temperature that are not produced during culture at normal temperature, and we designated these substances heat-shock metabolites (HSMs).^5^ We also discovered new compounds with unique biological activities and suggested the importance of temperature change in the production of SMs in actinomycetes.^6,7^ However, the physiological significance and reason that actinomycetes produce HSMs upon high-temperature cultivation have not been clarified.

**Figure 1:**
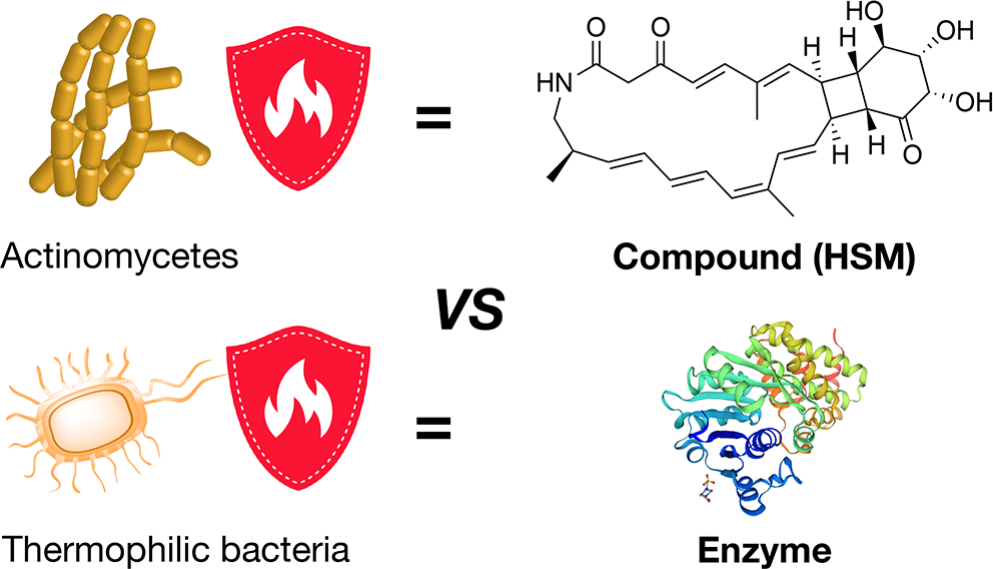
Conceptual diagram of the thermotolerance mechanism in microorganisms. 3D model of the protein was generated using SWISS-MODEL.

The actinomycetal strains we are currently studying are thermostable, which means they can grow at temperatures 10 to 15 °C above their optimum growth temperature. Thermophiles, which exist in hydrothermal vents, are microorganisms with optimal growth temperatures of 50–80 °C or higher, and hyperthermophiles are microorganisms with optimal growth temperatures of 80–110 °C.^8^ The thermotolerance of thermophilic bacteria is derived from a special enzyme that is stable at high temperatures.^9^ However, these enzymes do not function well at temperatures below 40 °C. We therefore hypothesized that thermotolerant actinomycetes have a mechanism that enables them to utilize compounds such as HSMs to impart thermotolerance (Figure 1). The physiological significance of many SMs produced by actinomycetes remains unknown,^10^ and there are no reports of SMs that play a role in thermotolerance in all organisms.

**Figure 2:**
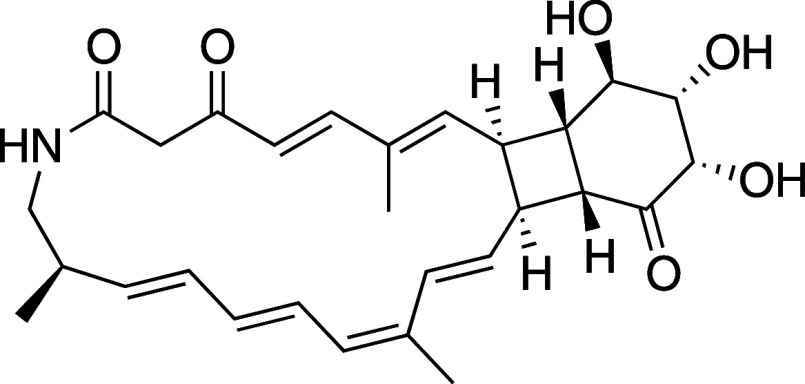
Structure of streptolactam D.

In this study, we isolated and determined the structure of a new compound, streptolactam D, as an HSM from thermotolerant *Streptomyces* sp. JA74, which was selected from a phylogenetic viewpoint (Figure 2). This compound was found to localize in the cell membrane of strain JA74 and stabilize the membrane’s fluidity under high-temperature conditions, thereby conferring thermotolerance. This is the first report demonstrating utilization of an SM by actinomycetes for thermotolerance and proposes a new physiological function for macrolactam SMs.

## Results and Discussion

### Molecular Phylogenetic Analysis of Thermotolerant Actinomycetes

**Figure 3:**
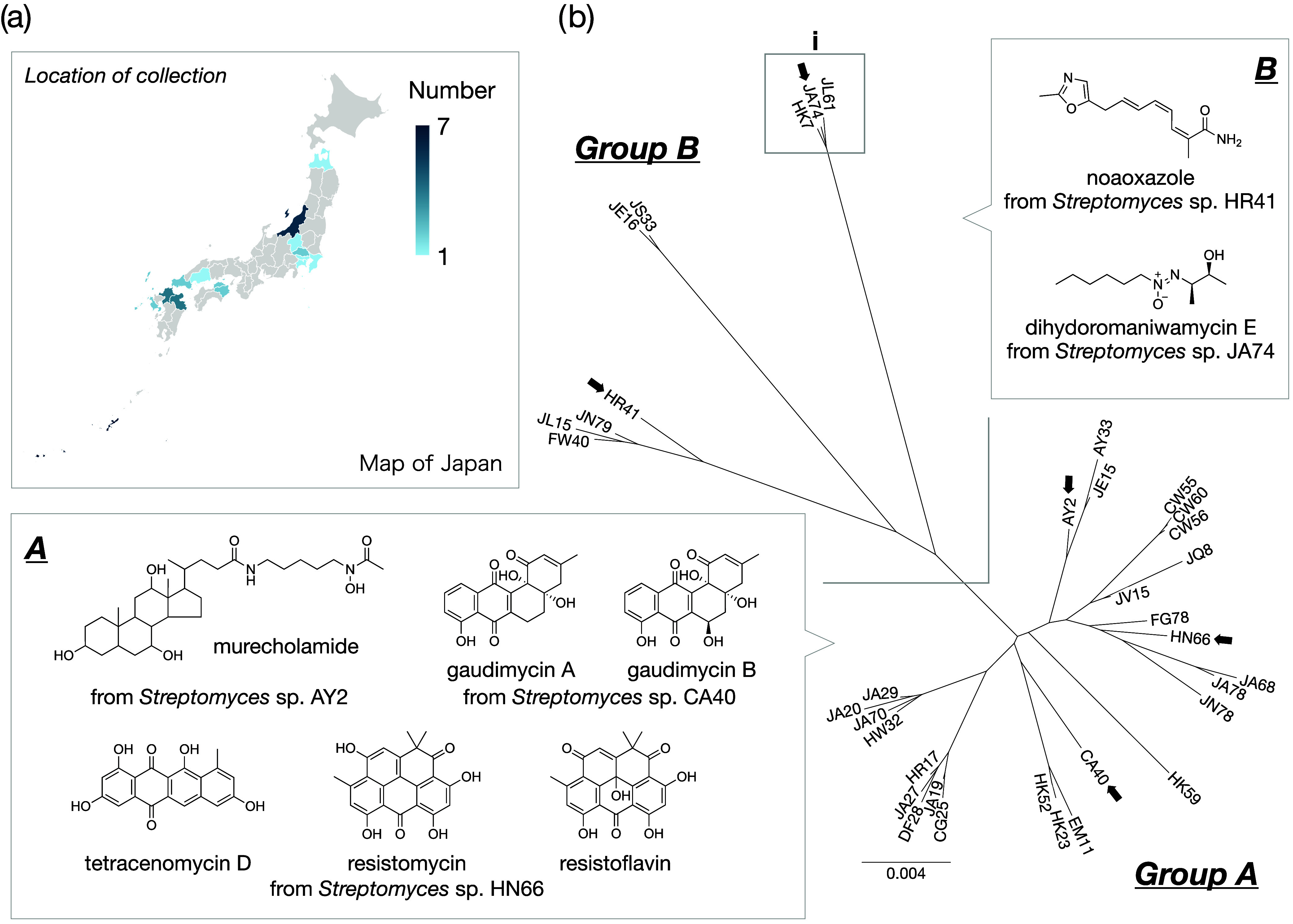
(a) Location and number of thermostable actinomycetes collected in Japan. (b) Molecular phylogenetic tree analysis of 36 thermotolerant *Streptomyces* strains and the representative structures of HSMs. Molecular phylogenetic tree was generated using Geneious Prime.

**Figure 4:**
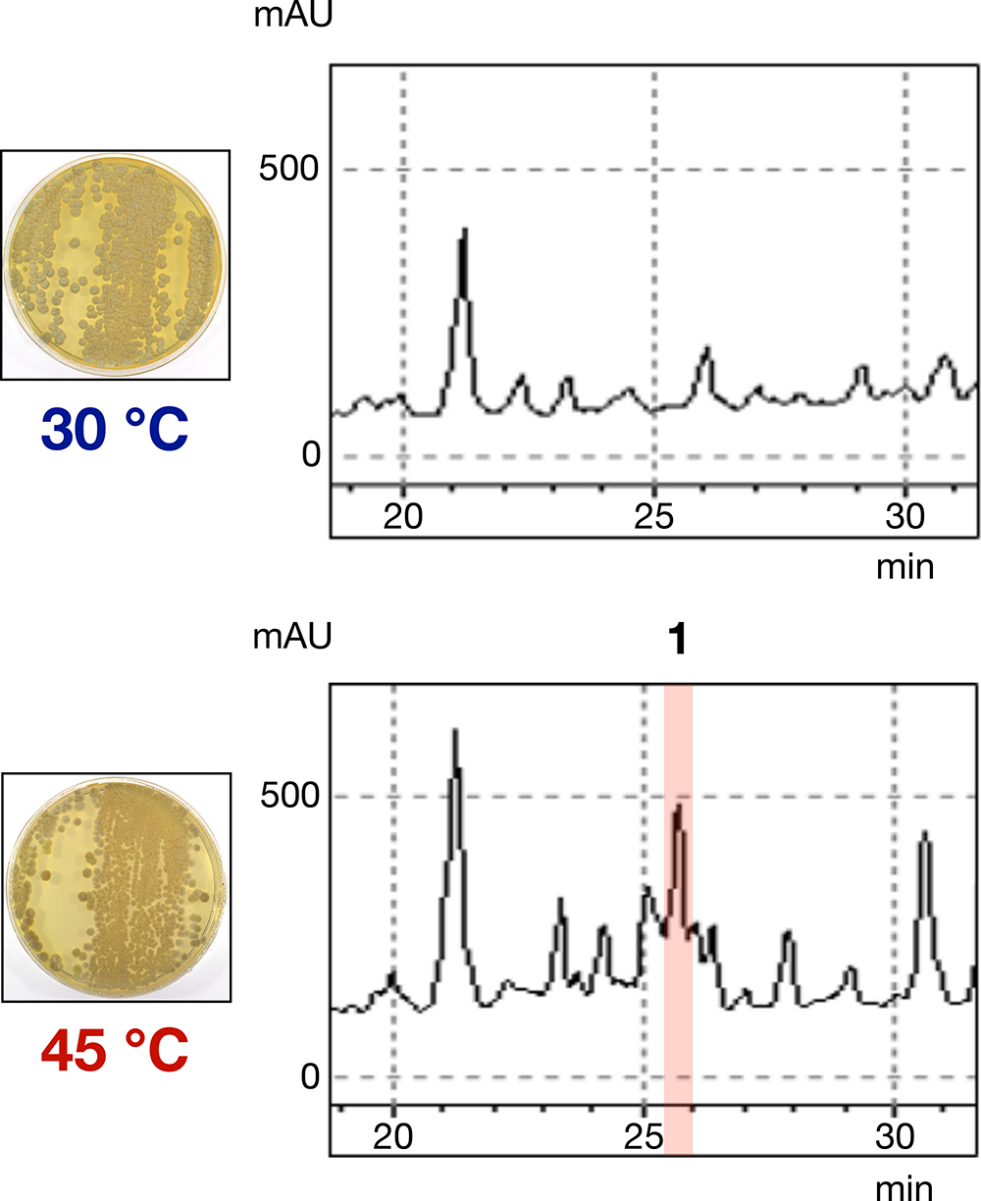
Analysis of metabolites of *Streptomyces* sp. JA74, which belongs to clade i in group B. HPLC charts were monitored at 290 nm.

In a previous study, we identified 57 strains of actinomycetes capable of growth at 45 °C from an in-house actinomycete library of 3160 strains (Figure 3a).^5^ Komaki’s group reported that even phylogenetically closely related species of actinomycetes have relatively different secondary metabolic capacities, suggesting that more distantly related species may produce unique SMs.^11^ Therefore, we attempted a molecular phylogenetic analysis based on 16S rRNA for all 57 strains of thermotolerant actinomycetes and identified 41 strains belonging to the genus *Streptomyces* (Table S1). A molecular phylogenetic tree was then constructed for the 36 HSM-producing strains, which indicated that the strains could be classified into two groups, A and B (Figure 3b). In our previous study, gaudimycins and resistomycins were produced by group A organisms,^5^ whereas new HSMs such as noaoxazole^6^ and maniwamycins^7^ were produced by group B organisms. In this study, we analyzed the metabolites of actinomycetes strains belonging to clade i in group B and found that three actinomycetes strains, HK7, JA74, and JL61, produce a common HSM with maximal UV absorbance at 290 nm (peak highlighted in red in Figure 4). Among the three strains, *Streptomyces* sp. JA74 exhibited a remarkable increase in HSM production, and we therefore attempted to isolate and determine the structure of the HSM.

### Isolation and Planar Structure Determination of HSM

*Streptomyces* sp. JA74 was cultured in ISP2 liquid medium under shaking conditions at 45 °C for 3 days, after which the entire culture broth was extracted with acetone and EtOAc. The resulting crude extract (1.4 g from 3.9 L) was fractionated by silica gel column chromatography, and the final purification was achieved using reversed-phase HPLC separation to yield compound **1** (0.7 mg). Because compound **1** was unstable, solvent removal by evaporation and each purification step were carried out in the dark.

Compound **1** was obtained as a pale-yellow powder. The molecular formula, deduced from a pseudomolecular ion at *m*/*z* 504.2359 [M + Na]^+^ in HR-ESI-TOF-MS analysis, was C_28_H_35_NO_6_, which corresponded to 12 degrees of unsaturation. Analysis of the IR spectrum indicated the presence of exchangeable proton(s) (3344 cm^–1^) and carbonyl (1681 cm^–1^) functional groups, which was corroborated by ^1^H and ^13^C NMR signals for one broad singlet exchangeable proton (δ_H_ = 8.67) and one carboxyl or amide carbon (δ_C_ = 165.8). ^13^C NMR and HMQC spectral data confirmed the presence of 28 carbons assignable to two ketone carbons, one carboxyl or amide carbon, and 12 olefinic or aromatic carbons, of which 10 were proton-bearing and 8 were sp^3^ methines, of which three were oxygenated or nitrated, respectively, 2 were sp^3^ methylene, and 3 were methyl groups (Table 1).

**Table 1:** ^1^H and ^13^C NMR Data for Streptolactam D (**1**) in Pyridine-*d*_5_

no.	δ_C_^a^	δ_H_ (*J* in Hz)^b^	HMBC^c^
1	165.8, C		
2	52.8, CH_2_	3.65, 3.78 (1H, m)	1, 3
		3.78 (1H, d, 12.6)	
3	193.5, C		
4	122.5, CH	6.74 (1H, d, 15.5)	3, 6
5	148.5, CH	7.54 (1H, t, 15.5)	3, 7, 26
6	133.9, C		
7	147.3, CH	6.06 (1H, d, 9.7)	5, 26
8	41.7, CH	4.53 (1H, t, 9.7)	
9	50.3, CH	3.50 (1H, t, 9.7)	11
10	70.9, CH	4.50 (1H, m)	12, 14
11	80.8, CH	5.11 (1H, m)	
12	76.3, CH	5.54 (1H, m)	13
13	212.8, C		
14	48.5, CH	3.38 (1H, dd, 3.2, 9.7)	
15	42.5, CH	4.15 (1H, m)	
16	130.6, CH	5.97 (1H, m)	17
17	132.1, CH	6.57 (1H, d, 15.8)	15, 27
18	135.3, C		
19	128.7, CH	5.96 (1H, d, 11.2)	17
20	127.9, CH	6.15 (1H, dd, 11.7, 14.0)	
21	131.5, CH	6.11 (1H, dd, 11.7, 14.0)	
22	131.5, CH	6.06 (1H, dd, 10.9, 14.6)	
23	137.0, CH	5.29 (1H, dd, 8.3, 14.3)	
24	38.0, CH	2.79 (1H, m)	
25	46.2, CH_2_	2.79, 3.70 (2H, m)	
26	12.7, CH_3_	1.82 (3H, s)	5, 6, 7
27	20.6, CH_3_	1.86 (3H, s)	17, 18, 19
28	18.4, CH_3_	0.90 (3H, d, 6.0)	23, 24, 25
NH		8.67 (1H, brs)	

a 125 MHz.

b 500 MHz.

c From proton(s) to indicated carbons.

**Figure 5:**
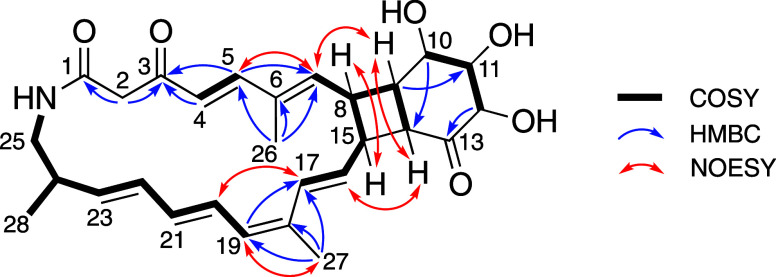
COSY, key HMBC, and NOESY correlations of streptolactam D (**1**).

Analysis of ^1^H–^1^H COSY spectra revealed four partial proton–proton connections from H4 to H5 (a), H7 to H9–H14 and H8–H15 to H17 (b), H10 to H12 (c), and H19 to H25–H28 (d) (Figure 5). Fragments (a) and (b) were joined by the HMBC correlations from a singlet methyl group H26 to C5, C6, and C7. This formed a fragment that was also combined with (c) and (d) based on the HMBC correlations from H9 to C11 and H10 to C14 for (c) and a singlet methyl group H27 to C17, C18, and C19 for (d). This combined fragment was further extended based on the HMBC correlations from one methylene group H2 (δ_H_ 3.65, 3.78, δ_C_ 52.8), H4, and H5 to ketone carbon C3 (δ_C_ 193.5), from H2 to carboxyl or amide carbon C1 (δ_C_ 165.8), and from H12 to ketone carbon C13 (δ_C_ 212.8), respectively. Furthermore, the NH group in the molecular formula was predicted to combine at C1 and C25 to form a macrolactam structure based on the ^1^H–^1^H COSY correlation between H25 and a broad singlet exchangeable proton (δ_H_ 8.67) presumed to be NH. In addition, based on the chemical shift values of C10 (δ_C_ 70.9), C11 (δ_C_ 80.8), and C12 (δ_C_ 76.3), the three remaining protons were predicted to correspond to hydroxyl groups in these positions. Finally, the remaining two degrees of unsaturation were satisfied by linking C13/C14/C15 and bonding a 4-membered ring fused to a 6-membered ring structure to the macrolactam structure.

The double-bond geometries between C4/C5, C16/C17, C20/C21, and C22/C23 were assigned as *E*, *E*, *E*, and *E* based on the respective *J* coupling constant values (Table 1). In addition, the geometries of C6/C7 and C18/C19 were determined as *E* and *Z* based on NOESY correlations between H5/H7, H17/H20, and H19/H27. Thus, the planar structure of **1** was determined to be a new macrolactam-type natural product with a unique 4-membered ring fused to a 6-membered ring structure. Streptolactam B was reported to be a geometric isomer of **1**,^12^ and we therefore designated the new HSM streptolactam D.

### Structure Determination of Streptolactam D

To determine the stereochemistry of streptolactam D (**1**), we first identified the NOESY correlations among H7/H9, H8/H15, H9/H14, and H14/H16. These correlations indicated that the cyclobutane ring is *cis*-fused to the 6-membered ring and the macrocycle (Figure 5). However, the remaining NOESY correlations did not provide sufficient information to determine the relative stereochemistry of the C10, C11, C12, and C24 positions. In addition, the use of direct structure determination methods, including chemical transformation methods, was not possible because compound **1** is unstable and difficult to obtain in amounts sufficient for these analyses.

**Figure 6:**
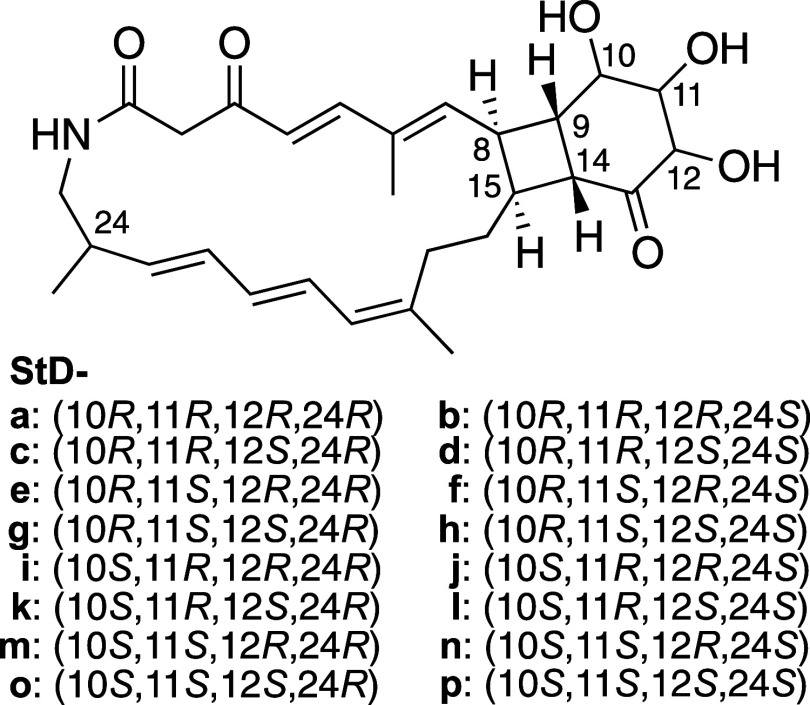
Sixteen candidate structures of streptolactam D (**1**).

We next conducted DP4+ analysis to determine the remaining relative stereochemistry of streptolactam D (**1**). DP4+ analysis employs density functional theory (DFT)-based NMR calculations and statistical methods to generate probability values for candidate structures in order to identify the most plausible true structure.^13,14^ We designed 16 candidate models, **StD-a–p** (Figure 6), in which the cyclobutane moiety was fixed to the *cis*-fused configuration, (8*S*,9*S*,14*R*,15*S*), and DP4+ analysis was then performed using chemical shifts estimated from DFT calculations and measured shifts in pyridine-*d*_5_. The analysis identified **StD-g** as the most plausible candidate structure (Table 2). Furthermore, the mean absolute error and maximum error of NMR calculations for candidate structure **StD-g** were reasonable values for the true structure of compound **1** (Table S2). Considering that the relative configuration is the same as that of the known streptolactam B, we determined that the relative stereochemistry of **1** is (8S*,9S*,10R*,11S*,12S*,14R*,15S*,24R*).

**Table 2:** DP4+ Probabilities for 16 Candidate Models, **StD-a**-**p**

StD-	a	b	c	d	e	f	g	h
DP4+	0%	0%	2.3%	0%	0%	0%	97.6%	0%

StD-	i	j	k	l	m	n	o	p
DP4+	0.1%	0%	0%	0%	0%	0%	0%	0%

**Figure 7:**
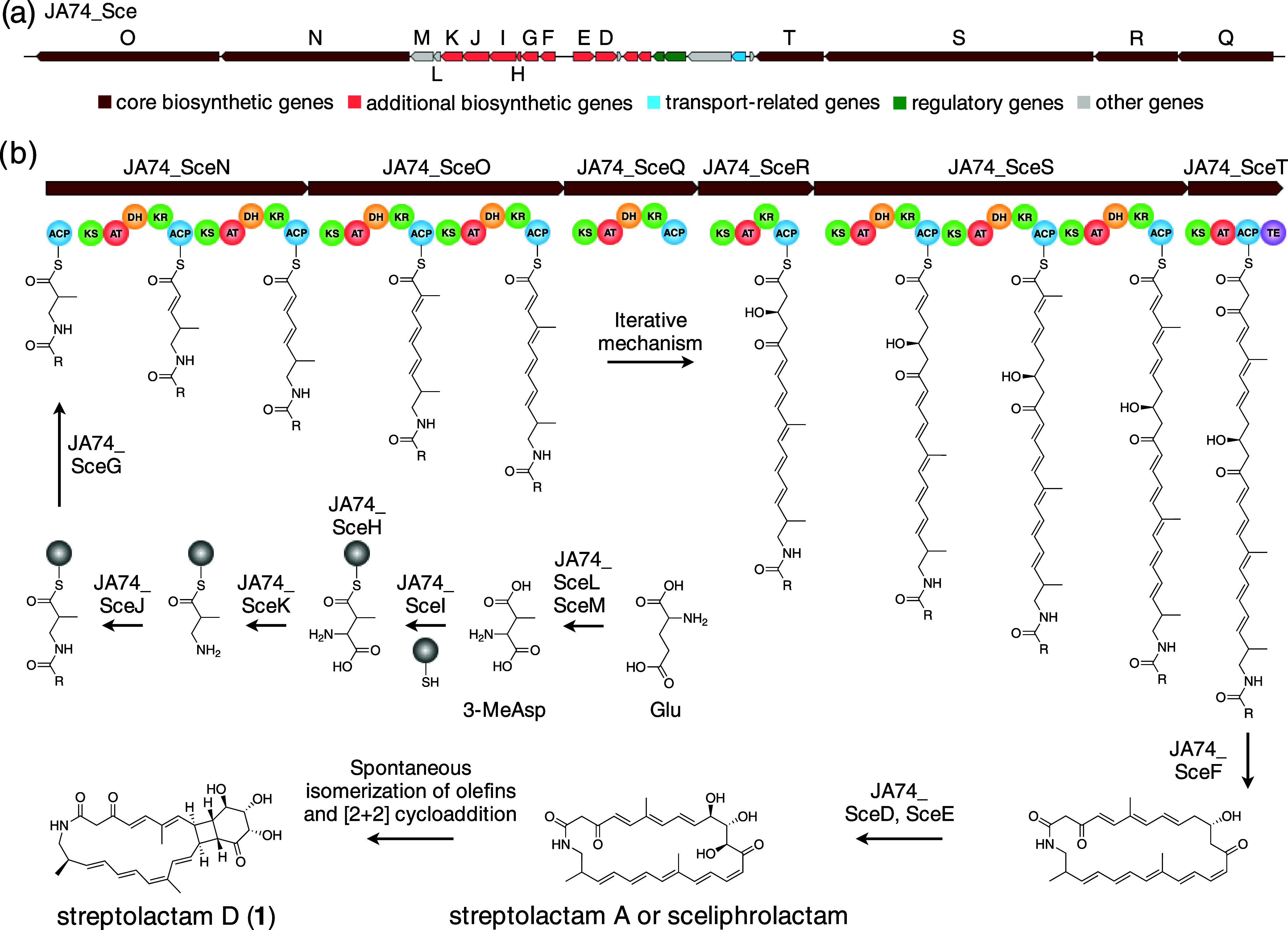
Putative pathway of the biosynthesis of streptolactam D (**1**). (a) Organization of the biosynthetic gene cluster of compound **1**. (b) Putative biosynthetic pathway for **1**. ACP, acyl carrier protein; AT, acyltransferase; DH, dehydratase; KR, ketoreductase; KS, ketosynthase; TE, thioesterase. Panel (a) was created based on the results of antiSMASH.

Having determined the relative stereochemistry of streptolactam D (**1**), we then focused on determining its absolute configuration (i.e., whether compound **1** is **StD-g** or *ent*-**StD-g**) using draft genome analysis and ECD calculations. A draft genome analysis of streptolactam D (**1**)-producing strain JA74 was performed using antiSMASH to identify the biosynthetic genes.^15^ This analysis confirmed the presence of a biosynthetic gene cluster exhibiting high homology to the biosynthetic gene clusters for sceliphrolactam and tripartilactam (Figure 7a and Table S3).^16,17^ Tripartilactam is produced from streptolactam A, a stereoisomer of sceliphrolactam. Therefore, this confirmed cluster is expected to mediate the biosynthesis of **1**. This cluster (JA74_*sce* cluster), spanning approximately 71.0 kb, was found to contain 24 open reading frames, including six polyketide synthase (PKS) genes, seven β-amino acid starter unit biosynthetic genes, two cytochrome P450 monooxygenase genes, three transport and regulatory genes, and several other genes. The biosynthesis of **1** is expected to be initiated by the incorporation of an amino-protecting 3-aminoisobutyric acid unit as a starter unit, similar to the biosynthesis of sceliphrolactam and tripartilactam (Figure 7b). Subsequently, the polyketide chain is extended by 10 extender modules and then macrocyclized by the thioesterase domain of JA74_SceT. In addition, two cytochrome P450 monooxygenases (JA74_SceD and JA74_SceE) catalyze the formation of hydroxy groups at C10 and C12 to generate streptolactam A or sceliphrolactam. Finally, a [2 + 2] cycloaddition reaction then takes place to complete the biosynthesis of **1**.

In general, the stereochemistry of the hydroxyl group derived from PKS can be determined by analyzing the sequence of the ketoreductase (KR) domain.^18^ The hydroxyl groups at C10 and C12 of sceliphrolactam are thought to be introduced by respective P450 enzymes.^19^ However, because the hydroxyl group at C11 is thought to be generated by the activity of PKS, we analyzed the sequence of the KR domain in JA74_SceR, which controls the absolute configuration at C11. The key sequence of the KR domain perfectly matched that of sceliphrolactam (Figure S7), indicating that the absolute configuration at C11 is *S*.

**Figure 8:**
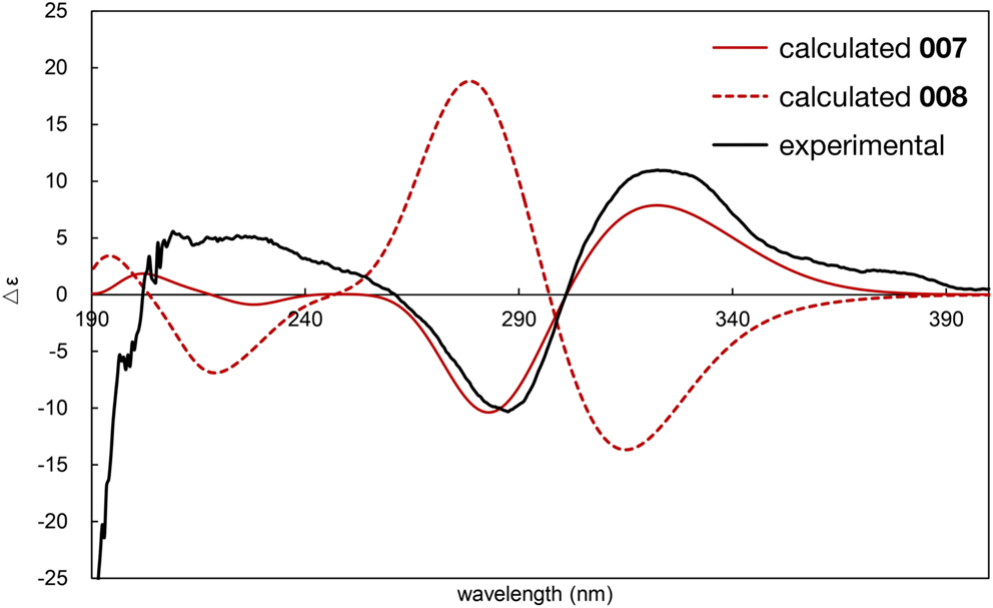
Experimental and calculated ECD spectra of streptolactam D (**1**) in MeOH. Red solid line: calculated spectrum of **StD-g**; red dashed line: calculated spectrum of *ent*-**StD-g**; black solid line: actual measured spectrum.

ECD spectrum simulations were also performed to confirm the absolute configuration of streptolactam D (**1**). ECD was calculated by using time-dependent DFT (TDDFT) at the ωB97xD/def2-TZVP level of theory, and solvent effects were accounted for using IEFPCM (MeOH). This analysis indicated that the spectrum of compound **1** was more consistent with the calculated spectrum of **StD-g** than with the spectrum of *ent-***StD-g** (Figure 8). Based on these collective results, the absolute stereochemistry of **1** was determined to be (8*S*,9*S*,10*R*,11*S*,12*S*,14*R*,15*S*,24*R*).

### Thermotolerance-Promoting Activity of Streptolactam D

**Figure 9:**
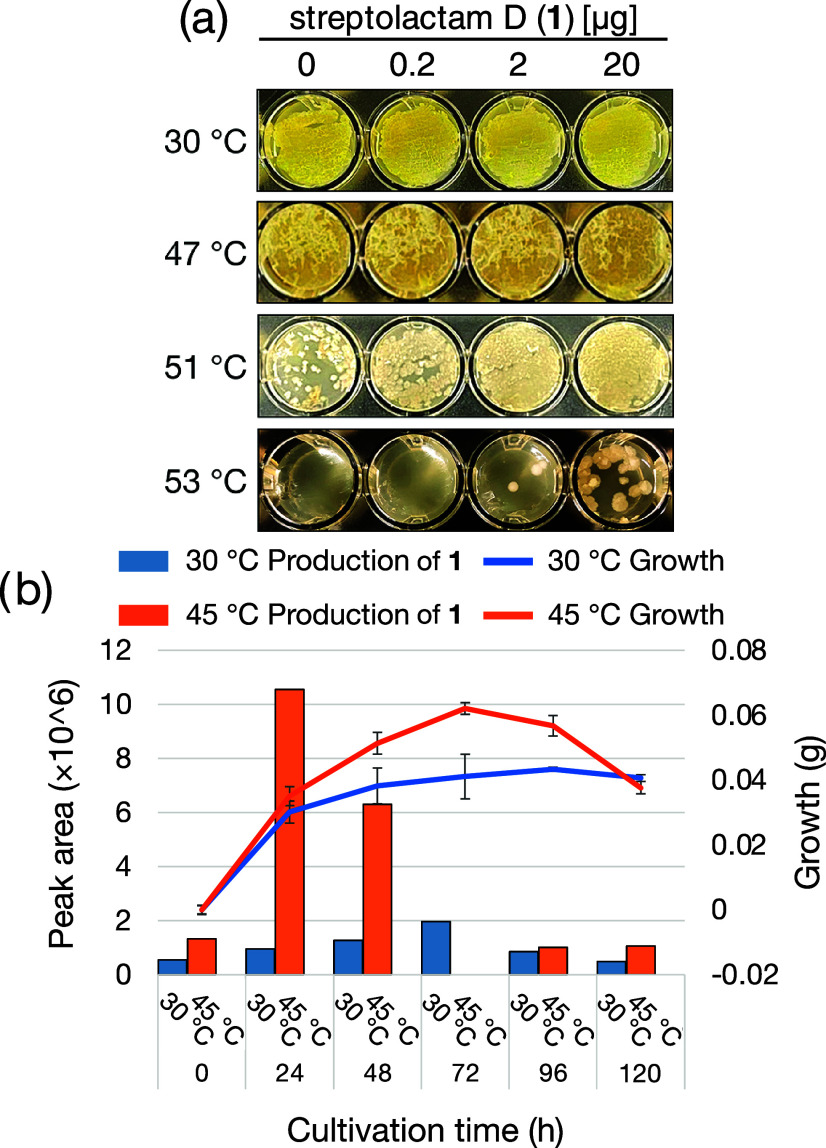
Effect of streptolactam D (**1**) on the growth of the producing strain JA74. (a) Observation of growth at various temperatures on ISP2 agar medium by treatment of **1** for 48 h. (b) Successive analysis of growth rate and the amount of **1** produced in ISP2 liquid medium.

The effect of streptolactam D (**1**) on the growth of strain JA74 on the ISP2 agar medium at a high temperature was also evaluated. Growth of strain JA74 deteriorated at 51 °C in the absence of exogenous **1**, and cells became nonviable at 53 °C. Surprisingly, compound **1** promoted the growth of strain JA74 at 51 °C and allowed growth at 53 °C, the growth-limiting temperature (Figure 9a). Successive analyses of growth rate and the amount of **1** produced in ISP2 liquid medium showed that strain JA74 entered a logarithmic growth phase at 12–24 h of cultivation, which was accompanied by a significant increase in the production of **1** under high-temperature conditions (Figure 9b). Interestingly, the abundance of **1** tended to decrease in the later stages of cultivation, likely due to its chemical instability, and the growth of strain JA74 tended to deteriorate accordingly.

These results suggest that compound **1** plays an important role in the growth of strain JA74 under high-temperature conditions. In the subsequent discussion, we express this effect as a “thermotolerance-promoting activity”.

### Analysis of the Subcellular Localization and Phenotypic Effects of Streptolactam D

**Figure 10:**
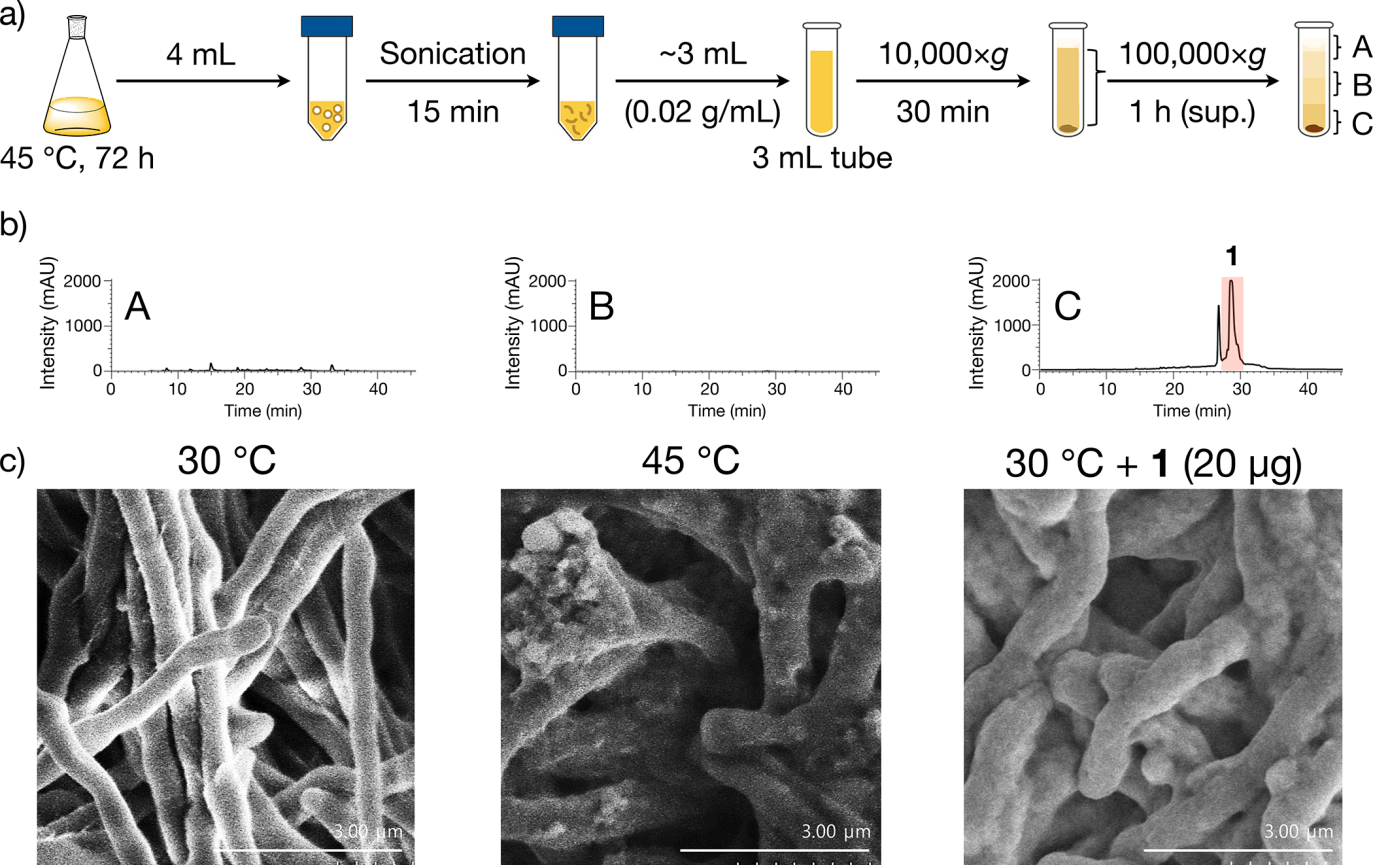
Analysis of subcellular localization of streptolactam D (**1**). (a) Membrane fractionation using ultracentrifugation. (b) HPLC analysis monitored at 290 nm for each fraction. (c) Scanning electron microscopy (SEM) observation of mycelia formed on ISP2 agar medium.

How streptolactam D (**1**) confers thermotolerance was also investigated. First, we analyzed the subcellular localization of compound **1**, focusing on a report of a compound with a macrolactam structure inserting into the cell membrane.^20^ Membrane fractionation of strain JA74 was performed by using ultracentrifugation and HPLC analysis of each resulting fraction. The data showed that **1** was present in the membrane fraction (Figure 10a,b). From the respective *R*_f_ values of TLC analyses, it was inferred that the membrane fraction contained various lipid components such as ceramides, fatty acids, and cardiolipin (Figure S8),^21^ components reported as membrane lipids in *Streptomyces coelicolor* A3(2) and M145.^22^

Because **1** was found to localize in the membrane fraction, we then examined the phenotypic changes using scanning electron microscopy. First, we compared the phenotype of strain JA74 at 30 and 45 °C and observed that the mycelia tended to be thicker and shorter at 45 °C than at 30 °C (Figures 10c and S9).

The insertion of cholesterol, a common lipid component of animal cells, imparts thickness to the cell membrane.^23^ When strain JA74 was treated with **1** and cultured at 30 °C, the phenotype was similar to that observed in the culture at 45 °C. This suggests that **1** localizes to the cell membrane in a cholesterol-like manner.

### Effect of Streptolactam D on Membrane Stability

**Figure 11:**
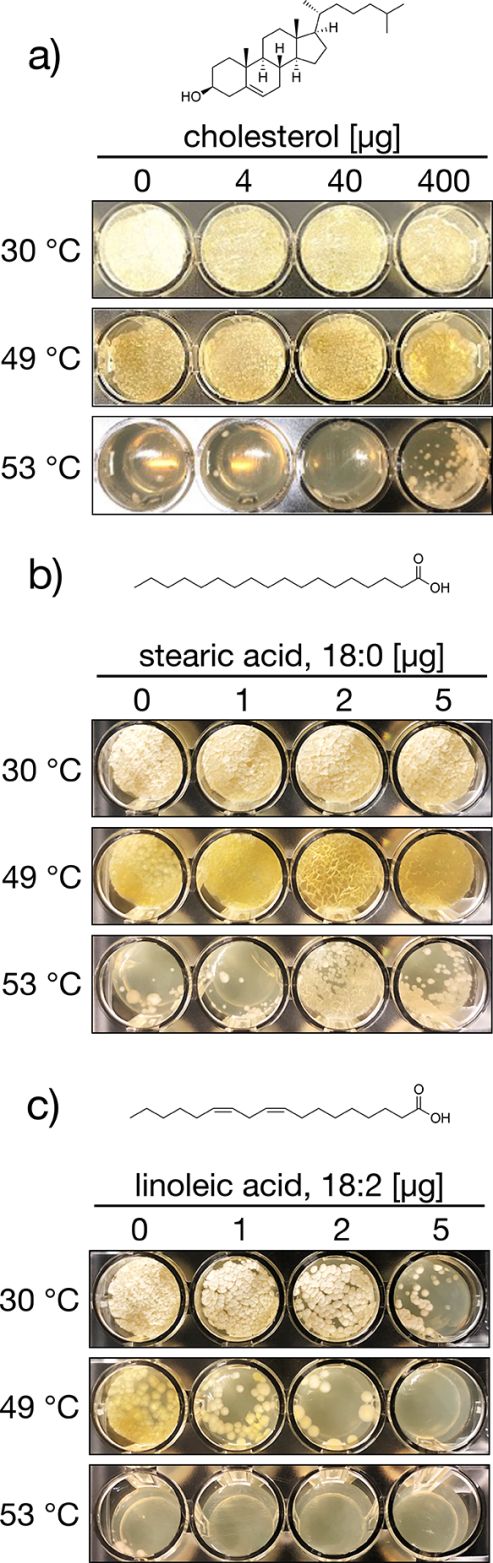
Effect of substances that impact cell membrane stability: (a) Cholesterol, (b) stearic acid, a saturated fatty acid, (c) linoleic acid, an unsaturated fatty acid. Each fatty acid was treated at various concentrations for 48 h.

In general, the composition of the cell membrane is sensitive to changes in temperature; thus, thermal stress can destabilize membrane fluidity.^24^ Therefore, we examined whether streptolactam D (**1**) confers thermotolerance upon straining JA74 by stabilizing the cell membrane. Cholesterol contributes to the stabilization of fluidity due to insertion into the cell membrane.^25^ Therefore, we first evaluated the effect of cholesterol on the growth of strain JA74. Cholesterol promoted the growth of JA74 at high temperatures, similar to **1** (Figure 11a). We also evaluated the effect of saturated and unsaturated fatty acids on the growth of JA74, as these fatty acids are known to affect cell membrane stability.^26^ Saturated fatty acids enabled growth at the growth-limiting temperature (53 °C), whereas unsaturated fatty acids, which destabilize cell membranes, suppressed growth at higher temperatures (Figure 11b,c). Therefore, the thermotolerance of strain JA74 was attributed to stabilization of the cell membrane.

**Figure 12:**
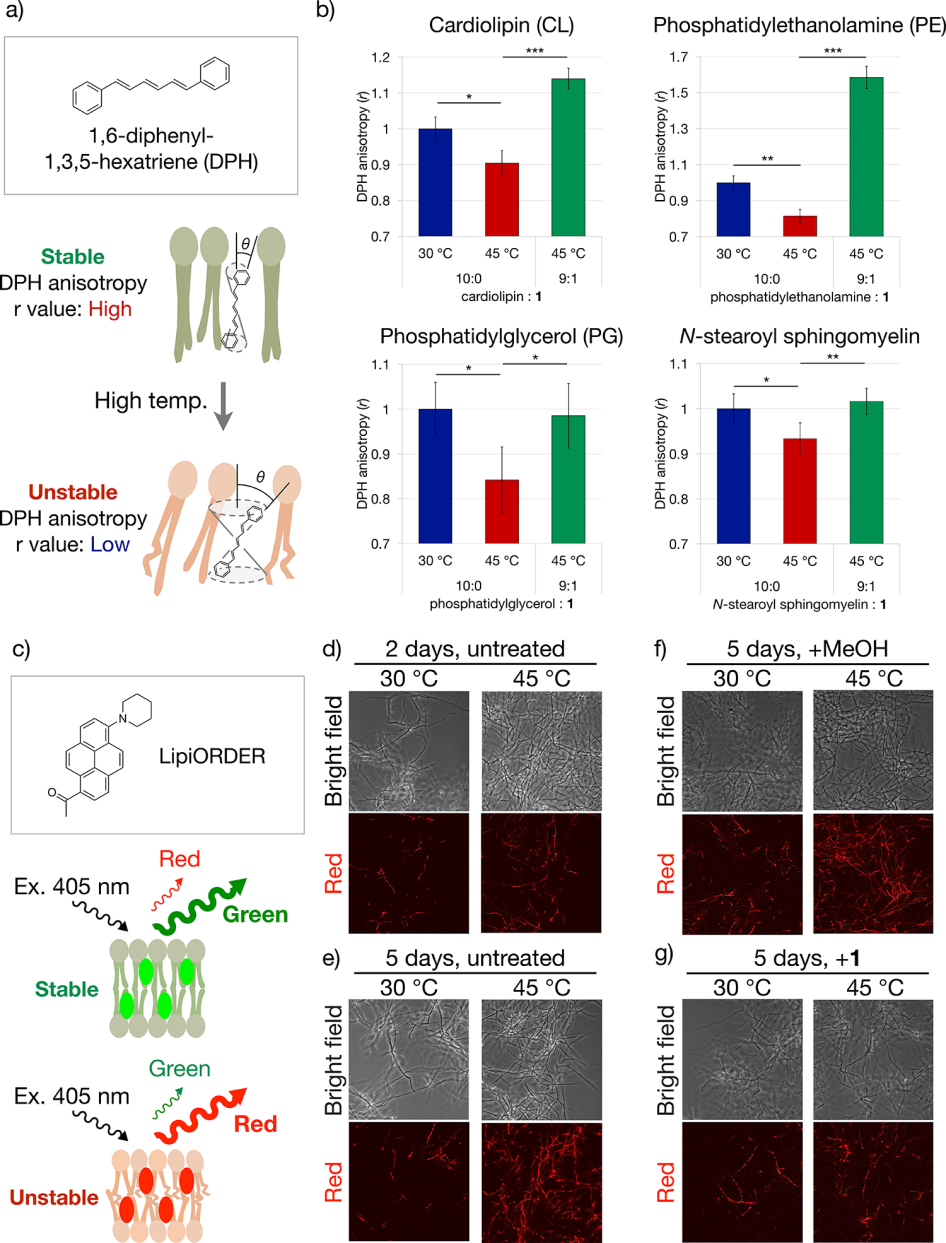
Observation of the membrane phase state of streptolactam D (**1**)-producing strain using 1,6-diphenyl-1,3,5-hexatriene (DPH) and LipiORDER. (a) Schematic diagram showing the relationship between the fluorescence anisotropy value (r) and cell membrane fluidity. (b) Effect of **1** on fluorescence anisotropy values of various membrane lipids, such as cardiolipin (CL), phosphatidylethanolamine (PE), and phosphatidylglycerol (PG), using DPH. DPH was added at 1 mol % of the mixture of each lipid and **1**. All data presented as mean ± SD (*n* = 3; *p < 0.05, **p < 0.01, ***p < 0.001; one-tailed Student’s *t* test). (c) Schematic diagram showing the mechanism of LipiORDER. (d, e) Observation of membrane phase state at each incubation time. Upper: 2 days; lower: 5 days. (f, g) Observation of the effect of **1** (2 μg) on the phase state of the membrane. Upper: without addition of **1**; lower: with addition of **1**.

Finally, we examined whether **1** is inserted into the cell membrane and contributes to stabilization. A verification experiment was carried out at the *in vitro* level. Poojari et al. analyzed changes in membrane fluidity by comparing the fluorescence anisotropy values (*r*) of 1,6-diphenyl-1,3,5-hexatriene (DPH) in artificial membrane systems (Figure 12a).^27^ Under high-temperature conditions, this membrane becomes unstable, and the value of *r* decreases. Thus, we examined the effect of **1** on the mobility of various lipids contained in the cell membrane of actinomycetes, such as cardiolipin, phosphatidylethanolamine, and phosphatidylglycerol, in addition to *N*-stearoyl sphingomyelin, which is often used in such evaluations, using DPH (Figure 12b). In the absence of **1**, a decrease in fluorescence anisotropy was observed at high temperature (ratio of each lipid to **1** = 10:0). By contrast, the addition of **1** tended to reverse this decrease in fluorescence anisotropy (ratio of each lipid to **1** = 9:1). These results indicated that **1** mitigates the reduction in the ordering effect of lipid membranes in high-temperature environments.

A similar experiment was performed at the *in vivo* level. Here, we attempted to observe the phase state of the cell membrane by imaging using LipiORDER, an environmentally responsive fluorescent dye (Figure 12c).^28^ LipiORDER exhibits green fluorescence (fluorescence maximum ∼510 nm) in the liquid ordered phase due to tight packing, whereas in the liquid disordered phase, in which the density is low, LipiORDER exhibits red fluorescence (fluorescence maximum ∼575 nm) due to a long wavelength shift. From successive analyses of the growth rate and productivity, the production of **1** tended to increase in the early stages of culture and decrease in the later stages (Figure 9b). Therefore, we conducted imaging analyses of the membrane phase state in the early and late stages of culture (2 or 5 days). No temperature-related changes in the intensity of red fluorescence were observed in the early stage (2 days) of culture when **1** increased (Figure 12d). By contrast, it increased at high temperature in the later stage (5 days) of culture, when **1** decreased (Figure 12e). These results suggested that the cell membrane is destabilized in the absence of **1** under high-temperature conditions. We then verified that **1** reverses this cell membrane instability in the late stage of the culture. Addition of **1** to the culture medium stabilized cell membrane fluidity in the late stages of culture, as it suppressed the increase in the intensity of red fluorescence at a high temperature (Figure 12f,g). Collectively, these data suggest that **1** inserts into the cell membrane of strain JA74 and provides stabilization.

## Conclusions

In this study, we isolated and determined the structure of a new compound, streptolactam D (**1**), produced by the thermotolerant *Streptomyces* sp. JA74, selected based on a phylogenetic perspective. Compound **1** was identified as a geometric isomer of streptolactam B, with a unique 4-membered ring fused to a 6-membered ring in the macrolactam structure. Streptolactam B is reportedly synthesized from the macrocyclic macrolactam streptolactam A in response to light irradiation.^12^ Draft genome and antiSMASH analyses of strain JA74 confirmed the presence of a biosynthetic gene cluster for sceliphrolactam,^19^ which has the same planar structure as streptolactam A. In strain JA74, **1** is thus thought to be produced nonenzymatically from streptolactam A during high-temperature culture. This structural change could be due to insertion into the cell membrane, but further analysis is needed to confirm this hypothesis.

Numerous natural products with a macrocyclic macrolactam structure that exhibit a variety of biological activities have been reported to date.^29^ However, the function of these endogenous compounds in producing organisms is poorly understood. The present study showed that **1** localizes to the cell membrane and stabilizes membrane fluidity under high-temperature conditions, which confers thermotolerance to strain JA74. When the cell membrane is unstable, the unsaturated fatty acid chains of phospholipids are bent and fluid in animal cells. In cholesterol, the hydrophilic hydroxyl group of cholesterol forms a hydrogen bond with the phosphate group of the phospholipid on the outside of the membrane, and the hydrophobic steroid ring is inserted between the fatty acid chains on the inside. In this way, the fatty acid chains become extended and stable, which is thought to enable cholesterol to adapt to changes in the environment. It is possible that **1** functions like cholesterol in the cell membrane, but the details require further investigation.

This is a novel physiological function of macrolactam-type SMs. However, production of **1** by strain JA74 tended to decrease in the later stages of culture. Strain JA74 also reportedly produces maniwamycins as HSMs,^7^ and these compounds promoted thermotolerance similar to that of **1** shown in Figure 9a (data not shown). These results suggest that the mechanism underlying the thermotolerance of strain JA74 is complex.

**Figure 13:**
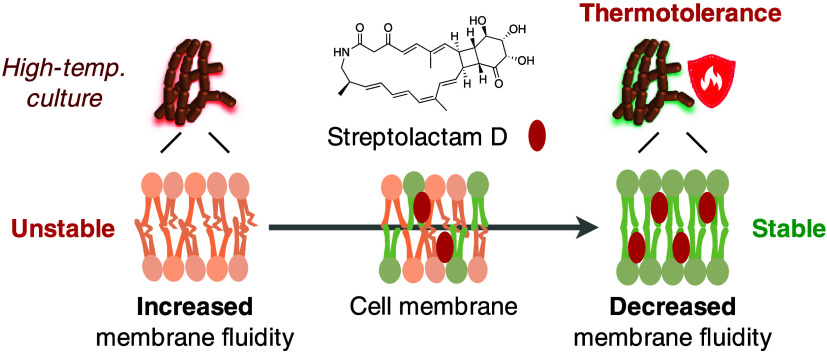
Mechanism of thermotolerance based on stabilization of the cell membrane by streptolactam D (**1**).

In summary, our analysis of the physiologic functions of HSMs revealed that strain JA74 produces **1** to support growth at high temperatures through self-insertion into the cell membrane, which provides stabilization of the membrane structure (Figure 13). This is the first report demonstrating that actinomycetes utilize an SM for thermotolerance and propose a new physiological function for macrolactam SMs.

Our results suggest that HSMs produced during high-temperature culture of actinomycetes promote thermotolerance of the producing organism. As the name “secondary metabolites” suggests, the endogenous functions of these compounds produced by actinomycetes are poorly understood. Elucidating the mechanisms regulating the production and physiologic functions of SMs in actinomycetes could facilitate the development of methods for activating silent genes. We thus plan to investigate why and how HSMs are produced in these organisms.

## Supporting Information Available

Experimental details; actinomycete strain information; NMR spectra, NMR chemical shifts calculation and biosynthetic gene cluster of streptolactam D (**1**); and supplemental figures as described in the text (PDF)
