# Supplementary material for: The Heat-Shock Metabolite Streptolactam D, Produced by High-Temperature Culture of Streptomyces sp. JA74, Promotes Thermotolerance via Self-Membrane Stabilization
Source: J Am Chem Soc. 2025 Apr 28;147(18):15676–85. doi: 10.1021/jacs.5c03026 (PMC12063178; doi:10.1021/jacs.5c03026)
Supplement: Supplementary file 1 — ja5c03026_si_001.pdf [file ja5c03026_si_001.pdf]

Supporting Information for

The Heat-Shock Metabolite Streptolactam D, Produced by High-Temperature Culture of *Streptomyces* sp. JA74, Promotes Thermotolerance via Self-Membrane Stabilization

Shun Saito[†*], Yurika Okumura[†], Sosuku Kataoka[†], Keisuke Fukaya[‡], Daisuke Urabe[‡], Midori A. Arai[†*]

[†]Department of Biosciences and Informatics, Keio University, 3-14-1 Hiyoshi, Kohoku-ku, Yokohama 223-8522, Japan
[‡]Biotechnology Research Center and Department of Biotechnology, Toyama Prefectural University, 5180 Kurokawa, Imizu, Toyama 939-0398, Japan

Table of Contents

Material and methods 3

Supplementary Tables

Table S1. Genetic analysis of thermotolerant actinomycetes based on the 16S rRNA sequence 9

Table S2. Results of chemical shift calculations and mean absolute error (MAE) in ppm for streptolactam D (**1**) 11

Table S3. Putative biosynthetic gene cluster of **1** 12

Supplementary Figures

Figure S1. ^{1}H NMR spectrum of streptolactam D (**1**) (500 MHz, Pyridine-d_5) 13

Figure S2. ^{13}C NMR spectrum of **1** (125 MHz, Pyridine-d_5) 14

Figure S3. ^{1}H-^{1}H COSY spectrum of **1** (500 MHz, Pyridine-d_5) 15

Figure S4. HMQC spectrum of **1** (400 MHz, Pyridine-d_5) 16

Figure S5. HMBC spectrum of **1** (500 MHz, Pyridine-d_5) 17

Figure S6. NOESY spectrum of **1** (500 MHz, Pyridine-d_5) 18

Figure S7. Sequence analysis of the KR domain 19

Figure S8. TLC analysis of the cell membrane fraction 20

References 21

Materials and methods

General experimental procedures

Optical rotations were measured using a JASCO P-1020 polarimeter (JASCO Corporation, Japan). UV spectrum was recorded on a Beckman DU530 UV/VIS spectrophotometer (Beckman Coulter, Inc., Brea, CA, USA). IR spectrum was recorded on a Bruker FT-IR ALPHA spectrometer (Bruker Corporation, Billerica, MA, USA). NMR spectra were obtained on a JEOL JNM-ECA500 spectrometer (JEOL Ltd., Japan) in Pyridine-d_5 and referenced to the residual solvent signals (δ_H 7.21, 7.57, 8.21, δ_C 123.5, 135.5, 149.5). HR-ESITOFMS spectrum was recorded on a LCT premier EX spectrometer (Waters Corporation, Milford, MA, USA). Silica gel 60 0.040-0.063 mm (KANTO CHEMICAL CO., INC., Japan) was used for silica gel column chromatography. HPLC separation was performed using a CAPCELL PAK C_{18} MGII packed column 20 × 250 mm (OSAKA SODA Co., Ltd., Japan) on a Hitachi ELITE LaChrom (Hitachi, Ltd., Japan). CD spectrum was measured by a Circular Dichroism spectrometer J-1100 (JASCO Corporation).

16S rRNA Gene Sequencing

Actinomycete strains were isolated from soil collected in Japan and stored in 20% glycerol. Genomic DNA of each strain was extracted using a NucleoSpin® Microbial DNA kit (Macherey-Nagel GmbH & Co. KG, Dueren, Germany). The 16S rRNA gene was amplified using universal primers (27F: 5'-AGAGTTTGATCCTGGCTCAG-3', 515F: 5'-GTGCCAGCMGCCGCGGTAA-3', 907R: 5'-CCGTCAATTCMTTTRAGTTT-3', 1492R: 5'-GGTTACCTTGTTACGACTT-3'), and the DNA sequence of the obtained samples was analyzed by Fasmac Co., Ltd., Japan. The obtained sequence data were assembled using Geneious Prime (Bioinformatics Software for Sequence Data Analysis; Tomy Digital Biology Co., Ltd., Japan), and the genus of each strain was identified by BLAST search. The molecular phylogenetic tree was also generated using Geneious Prime.

Microorganism

Streptomyces sp. JA74 was isolated from soil collected in Oita, Japan. The strain was identified as a member of the genus *Streptomyces* on the basis of 100% similarity in the 16S rRNA gene sequence (1383 nucleotides; GenBank accession number LC647576) to *Streptomyces corchorusii* strain NBRC 13032 (NR_041098.1).

Fermentation

Strain JA74 growing on ISP2 agar medium [glucose 0.4%, yeast extract 0.4%, malt extract 1.0%, and agar 2.0% in distilled water (pH 7.3)] was inoculated into Erlenmeyer flasks containing 100 mL of ISP2 medium [glucose 0.4%, yeast extract 0.4% and malt extract 1.0% in distilled water (pH 7.3)]. The flasks were placed on a rotary shaker (160 rpm) at 30 °C for 2 days. Then, the seed culture (5 mL) was transferred into 500 mL Erlenmeyer flasks, each containing 100 mL of ISP2 medium. The flasks were placed on a rotary shaker (160 rpm) at 45 °C for 3 days.

Isolation

At the end of fermentation, 100 mL of acetone was added to each flask, and the flasks were allowed to shake for 1 h. The mixture was centrifuged at 6,000 rpm for 10 min, and the supernatant was evaporated to remove acetone. The aqueous layer was extracted by EtOAc and concentrated *in vacuo* to give 1.4 g of extract from 3.9 L culture. The extract was subjected to silica gel column chromatography with a gradient of $CHCl_3/CH_3OH$ mixture solvent (100:0, 100:1, 100:3, 100:5, 100:10, 100:30 and 0:100 v/v). The fraction 4 (100:5) was evaporated, and the residue was purified by preparative HPLC using an isocratic elution with 29% CH_3CN solution at 10 mL/min, yielding streptolactam D (**1**, 0.7 mg, t_R 95.0 min).

Streptolactam D (**1**): pale yellow powder; $[\alpha]^{16}_D$ +52.9 (*c* 0.0007, MeOH); UV (MeOH) λ_{max} (log ε) 226 (4.70), 290 (5.13), 326 (4.59) nm; IR (ATR) ν_{max} 3344, 2926, 1681 cm^{-1}; ^1H and ^{13}C NMR data, see Table 1; HR-ESITOFMS *m/z* 504.2359 $[M+Na]^+$ (calcd for $C_{28}H_{35}NNaO_6$, 504.2356).

Computational procedure

General information

Conformational sampling was performed with MacroModel implemented in the Maestro 11.7 software package.[1] All DFT-based calculations were performed with the Gaussian 16 Rev C.01 program.[2] Analysis of computational results for the conformers was performed using a Python multi-conformer library, ACCeL.[3] A part of these computations was conducted using the SuperComputer System, Institute for Chemical Research, Kyoto University.

Procedure for NMR calculation and DP4+ analysis of StD-a-p

Conformational sampling using 10,000 steps of the Monte Carlo Multiple Minimum with the OPLS4 force field afforded 212 conformational isomers of **StD-a** within the

5.0 kcal/mol energy window. The geometries were then refined using GFN2-xTB[4] afforded 13 conformational isomers within 3.0 kcal/mol energy window. The geometries of the obtained conformational isomers were then optimized at the M06-2X/6-31G(d) level of theory. Frequency calculations were carried out at the same level of theory to confirm the absence of imaginary frequencies. After eliminating duplicated structures, the energy evaluation of the geometries at the M06-2X/6-311+G(d,p) level of theory. The NMR was simulated using the GIAO method at the mPW1PW91/6-31+G(d,p) level of theory. The chemical shifts (δ_{calc}) were calculated according to the formula $\delta_{calc} = \sigma_0 - \sigma_x$, where σ_x is the shielding tensors of the low-lying conformers averaged by Boltzmann distribution and σ_0 is the shielding tensor of the reference standard (tetramethylsilane) calculated at the same level of theory as σ_x. According to the procedure, the chemical shifts of **StD-b**, **StD-c**, **StD-d**, **StD-e**, **StD-f**, **StD-g**, **StD-h**, **StD-j**, **StD-k**, **StD-l**, **StD-m**, **StD-n**, **StD-o**, and **StD-p** were calculated by using 12, 16, 15, 10, 16, 22, 19, 16, 19, 36, 53, 14, 28, 8, 15 conformers low-lying conformers at the DFT calculation from 226, 532, 696, 237, 286, 515, 564, 306, 317, 513, 654, 281, 388, 474, 480 OPLS4-optimized structures, respectively. The results of the calculated chemical shifts are summarized in Table S2. For the analysis of DP4+, we used the same protocol, with the optimization using the B3LYP/6-31G(d) level of theory.

Procedure for ECD calculation of StD-g

Conformational sampling using 10,000 steps of the Monte Carlo Multiple Minimum with the OPLS4 force field afforded 226 conformational isomers of **StD-g** within the 4.0 kcal/mol energy window. The geometries of the obtained conformational isomers were then optimized at the M06-2X/6-31G(d) level of theory. Frequency calculations were carried out at the same level of theory to confirm the absence of imaginary frequencies. After eliminating duplicated structures, the energy evaluation of the geometries at the M06-2X/6-311+G(d,p) level of theory provided 25 low-lying conformers within 3.0 kcal/mol from the minimum Gibbs free energy. The geometries of the low-lying conformers were again optimized at the M06-2X/6-31G(d)-SMD(MeOH) level of theory. Frequency calculations were carried out at the same level of theory to confirm the absence of imaginary frequencies. After eliminating duplicated structures, the energy evaluation of the geometries at the M06-2X/6-311+G(d,p)-SMD(MeOH) level of theory provided 17 low-lying conformers within 2.0 kcal/mol from the minimum Gibbs free energy. The ECD was simulated by the TDDFT calculation of 25 excited states at the ωB97XD/def2-TZVP-IEFPCM(MeOH) level of theory. The spectra (half-width: 0.24

eV) were created by averaging spectra of the low-lying conformers according to the Boltzmann distribution and scaled to adjust the strength of the vertical axis.

Draft genome sequencing

Genome sequencing was performed on a NovaSeq 6000 sequencer (Illumina K.K., Japan). Shotgun and paired-end reads were assembled using Geneious Prime.

Assay of growth on agar medium

ISP2 agar medium (2 mL) was allocated to wells of 24-well plates. Test compound was added to each well at a volume of 10 or 20 µL. After drying of the solution, the wells were inoculated using a sterilized toothpick with strain JA74 growing on ISP2 agar medium (100-mm dish), and the plates were incubated at various temperatures.

Assay of growth in liquid medium

Strain JA74 growing on ISP2 agar medium (100-mm dish) was inoculated into Erlenmeyer flasks containing 100 mL of ISP2 liquid medium. The flasks were placed on a rotary shaker (160 rpm) and incubated at 30 °C for 2 days. Seed culture (5 mL) was then transferred into 500-mL Erlenmeyer flasks, each containing 100 mL of ISP2 medium. These flasks were placed on a rotary shaker (160 rpm) for 5 days. Culture medium was collected at 0, 24, 48, 72, 96, and 120 h of cultivation for generation of a growth curve and to monitor the production of streptolactam D (**1**). For growth curve generation, culture medium (1 mL) was collected three times, and the wet volume was measured at each time point. To examine the production of **1**, culture medium (0.5 mL) was collected and extracted using acetone and ethyl acetate. The extract was then analyzed by HPLC, and the peak area of **1** was determined at each time point.

Ultracentrifugation for cell membrane fractionation

Culture broth (4 mL) of strain JA74 was disrupted by continuous ultrasonication on ice for 15 min. The amount of bacteria was adjusted to 0.02 g/mL before ultrasonic disruption. After disruption, a sample (3 mL) was transferred to an ultracentrifuge tube and centrifuged (Hitachi, Ltd., Japan) at 10,000 × *g* for 30 min. The supernatant was transferred to a new ultracentrifuge tube and further centrifuged at 100,000 × *g* for 1 h. Three fractions were obtained: a white cloudy liquid appearing in the upper layer (fraction 1), a yellow transparent liquid portion in between (fraction 2), and a reddish-brown solid fraction that formed at the bottom (fraction 3). Finally, gradient HPLC analysis was performed under the following conditions: MeCN 15%→85% over

45 min, followed by MeCN 100% for 15 min.

TLC analysis of lipid components in the cell membrane

Cell membrane fraction 3 was mixed with 500 µL of ice-cold 0.15 M KCl solution. This mixture was transferred to glass test tubes with lids, and 3 mL of $CHCl_3$:MeOH (1:2) mixture was added. After vortexing for 3 min, the tubes were allowed to stand at room temperature for 10 min. Subsequently, 1.3 mL of a mixture of 0.2 M KCl and 0.1 N HCl and 1 mL of $CHCl_3$ was added, followed by vigorous vortex mixing for 3 min. The tubes were then centrifuged at 2,000 rpm for 5 min to remove the aqueous layer. For washing, 1 mL of $CHCl_3$ was added again, and the sample was vigorously vortexed for 30 sec and centrifuged at 2,000 rpm for 5 min to remove the supernatant and recover the organic layer at the bottom. The solvent was evaporated using blowing argon gas in a water bath at 40 °C. Subsequently, lipids adhering to the bottom wall of each glass test tube were dissolved using a mixture of $CHCl_3$:MeOH (2:1) (50 µL) to prepare the membrane fraction. A TLC plate (Merck & Co., Inc., Rahway, NJ, USA), 20 cm in length, was heated at 160 °C for 30 min and then allowed to cool to room temperature. The membrane fraction was developed in $CHCl_3$:MeOH:H_2O (14:6:1). Finally, the plate was sprayed with phosphomolybdic acid and allowed to cool, after which spots were detected by heating on a hot plate.

Analysis of phenotype by scanning electron microscopy

Strain JA74 was cultured on ISP2 agar medium at various temperatures. Colonies were cut into squares measuring 5-7 mm on each side, and excess agar was removed before thinly slicing the colonies. A solution of 4% osmium tetroxide (VIII) (Fujifilm Wako Pure Chemical Corp., Japan) diluted with an equal volume of Milli-Q water was placed in a light-tight container, and sliced colonies were steam-fixed overnight (or for 2 nights). The steam-fixed colonies were removed from the steam and affixed to sample holders using carbon tape. After drying in draft for 2.5-3 h, osmium coating was applied for 5 sec. Prepared samples were observed using an SU-70 field emission scanning electron microscope (Hitachi, Ltd., Japan).

Fluorescence anisotropy measurements

1,6-Diphenyl-1,3,5-hexatriene (DPH) (Santa Cruz Biotechnology, Inc., Dallas, TX, USA) 2 nmol and 200 nmol of each lipid, cardiolipin (CL; Sigma-Aldrich Co. LLC, St. Louis, MO, USA), phosphatidylethanolamine (PE; Fujifilm Wako Pure Chemical Corp.), and phosphatidylglycerol (PG; Sigma-Aldrich Co. LLC), were dissolved in 100 µL of

$CHCl_3$:MeOH (1:1). When streptolactam D (**1**) was added, the total amount of **1** together with each lipid was 200 nmol. The solvent was removed using an evaporator, and the sample was dried under high vacuum for 16 h. After drying, the samples were hydrated by adding 2 mL of Milli-Q water and incubated in a hot-water bath at 65 °C for 30 min, with vortexing every 10 min. After cooling to room temperature, each sample was transferred to a cell and measured using a Duetta fluorescence absorption spectrometer (Horiba Ltd., Japan). The measurement conditions were as follows: fluorescence anisotropy, excitation wavelength: 358 nm, fluorescence observation wavelength: 430 nm, temperature gradient increase rate 2 °C/min, measurement range 20-60 °C or 70 °C, number of measurement points 80 or 100, integration time 1 sec.

Analysis of the phase state of the cell membrane using confocal microscopy

Culture medium (1 mL) of strain JA74 incubated at 30 °C or 45 °C was centrifuged at 1,300 rpm for 2 min to collect bacterial cells. Next, 500 µL of HBSS(+) was added, and the supernatant was removed by centrifugation at 3,500 rpm for 2 min. As there was approximately 200 µL of bacterial cells, an equal volume (200 µL) of HBSS(+) with 2 µM LipiORDER® (Funakoshi Co., Ltd., Japan) was added. In addition, 2 µL of MeOH solution of 1 mg/mL streptolactam D (**1**) was added. This prepared sample was incubated at 30 °C or 45 °C for 10 min with stirring at 160 rpm and then centrifuged at 3,500 rpm for 2 min. After removal of the supernatant, the bacterial cells were placed on a slide glass and covered with a cover glass for observation. To compare green (500-550 nm) and red (550-650 nm) fluorescence resulting from excitation at 405 nm under confocal laser microscopy; Qdot525 (excitation wavelength: 405 nm, fluorescence detection wavelength range: 490-540 nm) was used for green fluorescence, and Qdot585 (excitation wavelength: 405 nm, fluorescence detection wavelength range: 570-620 nm) was selected for red fluorescence.

Table S1. Genetic analysis of thermotolerant actinomycetes based on the 16S rRNA sequence.

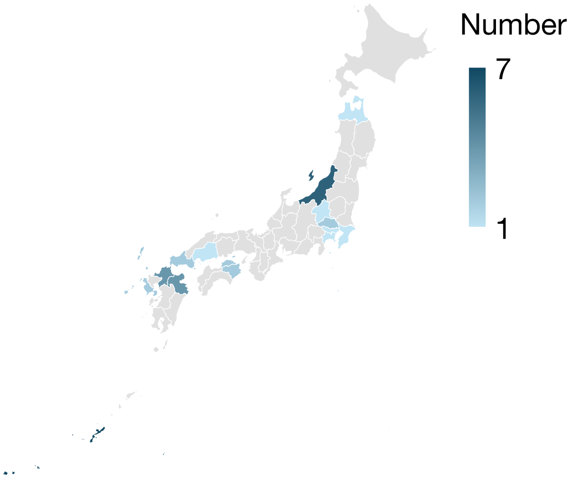

Name	Prefecture	Length (bp)	Genus (Results of blast analysis)	Ident. (%)
AY2	Kagawa	1392	*Streptomyces coeruleorubidus* strain KSRO2	100
AY6	Okinawa	1422	*Streptomyces variabilis* strain 13663R	100
AY33	Okinawa	1422	*Streptomyces tendae* strain KLK8	98.52
AY44	Okinawa	1406	*Streptomyces flavomacrosporus* strain UCRD31	99.93
AY46	Okinawa	100	*Streptomyces coeruleorubidus* strain KSRO2	100
AY58	Tokyo	1407	*Streptomyces naganishii* strain NRRL B-1816	99.79
CA40	Gunma	1403	*Streptomyces* sp. NBC_00557	98.50
CG25	Niigata	1424	*Streptomyces thermocarboxydus* strain H23A00036	99.93
CW55	Niigata	1408	*Streptomyces speibonae* strain CA47	99.93
CW56	Niigata	1408	*Streptomyces speibonae* strain CA47	99.86
CW60	Niigata	1408	*Streptomyces speibonae* strain CA47	99.86
DF28	Niigata	1395	*Streptomyces thermocarboxydus* strain EGI124	99.93
DF45	Niigata	1424	*Streptomyces thermocoprophilus* strain NBRC 100771	98.52
EM11	(Japan)	1406	*Streptomyces spinoverrucosus* strain NIIST A67	99.86
FG78	(Japan)	1423	*Streptomyces heilongjiangensis* strain FREP14	99.23
FW40	Kagawa	1408	*Streptomyces cuspidosporus* strain NBRC 12378	99.86
HK7	Nagasaki	1410	*Streptomyces corchorusii* strain NBRC 13032	99.93
HK23	Tokushima	1405	*Streptomyces glaucescens* strain S5-2	99.57
HK52	Tokushima	1406	*Streptomyces glaucescens* strain S5-2	99.72
HK59	Okinawa	1406	*Streptomyces misionensis* strain NBRC 13063	99.93
HN66	Okinawa	1487	*Streptomyces althioticus* strain NBC 01679	100

HR17	(Japan)	1408	*Streptomyces thermocarboxydus* strain H23A00037	99.93
HR41	(Japan)	1395	*Streptomyces* sp. RAI364	99.50
HW32	Kanagawa	1399	*Streptomyces asenjonii* strain R02	99.93
JA19	Fukuoka	1409	*Streptomyces thermocarboxydus* strain HD07	99.86
JA20	Fukuoka	1409	*Streptomyces cellulosae* SL2-2-R-9	99.72
JA27	Fukuoka	1409	*Streptomyces thermocarboxydus* strain H23A00037	99.93
JA29	Fukuoka	1424	*Streptomyces cellulosae* SL2-2-R-9	99.79
JA68	Oita	1407	*Streptomyces albogriseolus* strain H23A00039	99.93
JA70	Oita	1423	*Streptomyces cellulosae* SL2-2-R-9	99.93
JA74	Oita	1383	*Streptomyces corchorusii* strain NBRC 13032	100
JA78	Oita	1423	*Streptomyces albogriseolus* strain H23A00039	99.58
JE15	Yamaguchi	1426	*Streptomyces nigra* strain NBC_00206	99.44
JE16	Yamaguchi	1411	*Streptomyces albus* strain NBRC 15415	100
JL15	Saitama	1401	*Streptomyces cuspidosporus* strain NBRC 12378	100
JL61	Aomori	1406	*Streptomyces corchorusii* strain AUH-1	99.86
JN78	Saitama	1407	*Streptomyces* sp. strain MGB 2784	99.93
JN79	Chiba	1406	*Streptomyces ribosidificus* strain NBRC 13796	100
JQ8	Hiroshima	1405	*Streptomyces althioticus strain DSD2954*	99.86
JS33	Nagasaki	1407	*Streptomyces albus* strain NBRC 15415	100
JV15	Okinawa	1405	*Streptomyces albogriseolus* LRP	99.36

Table S2. Results of chemical shift calculations and mean absolute error (MAE) in ppm for **1**.

position	exp	StD-a	StD-b	StD-c	StD-d	StD-e	StD-f	StD-g	StD-h	StD-i	StD-j	StD-k	StD-l	StD-m	StD-n	StD-o	StD-p	|exp-StD-a|	|exp-StD-b|	|exp-StD-c|	|exp-StD-d|	|exp-StD-e|	|exp-StD-f|	|exp-StD-g|	|exp-StD-h|	|exp-StD-i|	|exp-StD-j|	|exp-StD-k|	|exp-StD-l|	|exp-StD-m|	|exp-StD-n|	|exp-StD-o|	|exp-StD-p|
14C	48.5	47.7	50.4	50.7	51.1	51.7	51.5	49.7	49.1	48.4	50.9	50.9	50.8	48.7	52.1	49.1	47.8	0.8	1.9	2.2	2.6	3.2	3.0	1.2	0.6	0.1	2.4	2.4	2.3	0.2	3.6	0.6	0.7
13C	212.8	208.5	207.1	209.9	209.6	204.8	204.7	212.2	211.6	209.1	208.0	209.1	209.1	206.4	205.3	211.2	210.5	4.3	5.7	2.9	3.2	8.0	8.1	0.6	1.2	3.7	4.8	3.7	3.7	6.4	7.5	1.6	2.3
12C	76.3	75.3	74.9	75.9	75.9	76.4	76.4	74.9	74.6	73.3	73.1	78.0	77.9	73.9	73.8	76.4	75.4	1.0	1.4	0.4	0.4	0.1	0.1	1.4	1.7	3.0	3.2	1.7	1.6	2.4	2.5	0.1	0.9
11C	80.8	76.9	77.4	78.9	78.4	78.3	78.4	79.5	79.6	77.9	78.2	77.5	77.7	78.9	79.4	77.5	77.2	3.9	3.4	1.9	2.4	2.5	2.4	1.3	1.2	2.9	2.6	3.3	3.1	1.9	1.4	3.3	3.6
10C	70.9	74.2	73.2	68.6	68.4	74.5	74.5	68.2	69.0	69.5	69.7	71.9	71.7	68.3	68.5	67.1	66.9	3.3	2.3	2.3	2.5	3.6	3.6	2.7	1.9	1.4	1.2	1.0	0.8	2.6	2.4	3.8	4.0
9C	50.3	48.8	50.1	49.0	47.3	47.8	47.6	52.6	50.5	42.8	45.0	44.0	44.9	42.2	44.0	50.1	47.0	1.5	0.2	1.3	3.0	2.5	2.7	2.3	0.2	7.5	5.3	6.3	5.4	8.1	6.3	0.2	3.3
8C	41.7	44.8	49.1	43.1	42.5	48.3	48.9	42.6	43.2	39.5	43.5	43.2	42.9	39.6	44.1	43.6	44.0	3.1	7.4	1.4	0.8	6.6	7.2	0.9	1.5	2.2	1.8	1.5	1.2	2.1	2.4	1.9	2.3
15C	42.5	47.0	48.3	44.7	47.6	48.7	49.0	45.7	49.7	47.9	49.9	47.5	45.8	48.8	49.2	45.4	49.6	4.5	5.8	2.2	5.1	6.2	6.5	3.2	7.2	5.4	7.4	5.0	3.3	6.3	6.7	2.9	7.1
16C	130.6	126.4	119.5	124.0	125.5	120.1	120.0	124.0	128.4	128.7	125.6	124.9	123.9	128.1	121.9	123.4	128.8	4.2	11.1	6.6	5.1	10.5	10.6	6.6	2.2	1.9	5.0	5.7	6.7	2.5	8.7	7.2	1.8
17C	132.1	130.2	132.6	134.1	130.3	133.2	133.4	134.6	131.6	128.8	132.5	130.1	131.5	129.0	133.6	134.4	130.9	1.9	0.5	2.0	1.8	1.1	1.3	2.5	0.5	3.3	0.4	2.0	0.6	3.1	1.5	2.3	1.2
18C	135.3	132.1	128.6	129.6	129.2	128.4	128.7	129.4	130.9	133.1	130.3	129.3	130.0	132.7	129.1	128.9	131.5	3.2	6.7	5.7	6.1	6.9	6.6	5.9	4.4	2.2	5.0	6.0	5.3	2.6	6.2	6.4	3.8
19C	128.7	129.5	131.4	130.1	130.5	131.4	131.4	129.8	129.6	128.8	130.0	130.6	130.3	129.0	131.0	130.4	129.5	0.8	2.7	1.4	1.8	2.7	2.7	1.1	0.9	0.1	1.3	1.9	1.6	0.3	2.3	1.7	0.8
20C	127.9	126.5	125.7	124.6	124.3	125.7	125.7	124.5	126.2	126.9	125.5	123.8	124.5	127.5	126.4	124.1	126.8	1.4	2.2	3.3	3.6	2.2	2.2	3.4	1.7	1.0	2.4	4.1	3.4	0.4	1.5	3.8	1.1
21C	131.5	132.2	133.4	133.1	134.9	134.1	133.5	132.9	133.2	131.6	133.3	134.9	133.0	131.8	133.1	133.5	132.5	0.7	1.9	1.6	3.4	2.6	2.0	1.4	1.7	0.1	1.8	3.4	1.5	0.3	1.6	2.0	1.0
22C	131.5	131.5	128.7	131.2	127.8	127.3	129.0	131.1	129.8	131.5	130.2	127.2	130.5	131.0	129.1	131.0	131.0	0.0	2.8	0.3	3.7	4.2	2.5	0.4	1.7	0.0	1.3	4.3	1.0	0.5	2.4	0.5	0.5
23C	137.0	136.8	141.6	137.7	141.9	140.5	141.3	138.0	138.5	135.9	139.9	138.9	136.8	136.3	140.8	138.4	137.3	0.2	4.6	0.7	4.9	3.5	4.3	1.0	1.5	1.1	2.9	1.9	0.2	0.7	3.8	1.4	0.3
24C	38.0	40.7	46.0	39.5	45.3	41.7	44.4	40.0	41.5	40.6	39.2	40.6	38.4	40.7	44.3	39.9	39.6	2.7	8.0	1.5	7.3	3.7	6.4	2.0	3.5	2.6	1.2	2.6	0.4	2.7	6.3	1.9	1.6
25C	46.2	48.0	47.8	48.5	47.7	46.0	48.0	48.4	48.1	48.0	49.9	45.1	46.6	47.9	48.3	48.3	48.5	1.8	1.6	2.3	1.5	0.2	1.8	2.2	1.9	1.8	3.7	1.1	0.4	1.7	2.1	2.1	2.3
27C	20.6	24.3	22.5	22.6	23.8	22.4	22.6	22.4	24.9	24.6	24.2	23.0	22.0	24.5	22.5	22.4	25.0	3.7	1.9	2.0	3.2	1.8	2.0	1.8	4.3	4.0	3.6	2.4	1.4	3.9	1.9	1.8	4.4
28C	18.4	19.9	19.4	19.7	19.5	15.9	19.5	19.6	19.1	20.0	19.2	17.4	18.4	19.5	18.8	19.6	19.3	1.5	1.0	1.3	1.1	2.5	1.1	1.2	0.7	1.6	0.8	1.0	0.0	1.1	0.4	1.2	0.9
1C	165.8	159.2	160.5	159.1	159.7	160.6	160.6	159.3	159.8	159.1	160.4	160.0	159.1	159.4	160.6	159.2	159.6	6.6	5.3	6.7	6.1	5.2	5.2	6.5	6.0	6.7	5.4	5.8	6.7	6.4	5.2	6.6	6.2
2C	52.8	55.2	51.4	52.8	51.9	51.6	50.5	52.5	51.8	55.7	47.7	52.3	49.8	55.3	50.5	52.9	51.6	2.4	1.4	0.0	0.9	1.2	2.3	0.3	1.0	2.9	5.1	0.5	3.0	2.5	2.3	0.1	1.2
3C	193.5	189.1	190.1	190.5	190.8	190.7	190.4	190.5	191.5	188.7	191.6	190.2	191.7	188.7	190.4	190.5	191.9	4.4	3.4	3.0	2.7	2.8	3.1	3.0	2.0	4.8	1.9	3.3	1.8	4.8	3.1	3.0	1.6
4C	122.5	120.0	121.4	122.0	121.8	121.7	122.3	120.9	123.2	119.7	125.5	121.9	122.9	120.4	122.3	119.8	124.2	2.5	1.1	0.5	0.7	0.8	0.2	1.6	0.7	2.8	3.0	0.6	0.4	2.1	0.2	2.7	1.7
5C	148.5	148.5	147.9	149.4	148.2	147.8	147.7	150.2	149.1	148.3	147.6	150.0	149.6	148.1	147.8	150.2	148.9	0.0	0.6	0.9	0.3	0.7	0.8	1.7	0.6	0.2	0.9	1.5	1.1	0.4	0.7	1.7	0.4
6C	133.9	133.9	139.9	133.2	136.5	139.8	140.0	133.7	135.3	132.0	139.4	134.6	134.2	133.6	140.3	131.2	134.2	0.0	6.0	0.7	2.6	5.9	6.1	0.2	1.4	1.9	5.5	0.7	0.3	0.3	6.4	2.7	0.3
7C	147.3	144.8	146.1	146.4	144.2	146.1	145.0	147.3	144.8	147.9	144.3	147.0	145.7	146.6	145.6	151.2	145.5	2.5	1.2	0.9	3.1	1.2	2.3	0.0	2.5	0.6	3.0	0.3	1.6	0.7	1.7	3.9	1.8
26C	12.7	14.5	17.1	15.2	18.2	17.6	17.5	15.2	18.2	14.5	17.8	18.2	17.2	14.9	17.5	14.4	18.2	1.8	4.4	2.5	5.5	4.9	4.8	2.5	5.5	1.8	5.1	5.5	4.5	2.2	4.8	1.7	5.5
8H	4.53	3.47	3.40	2.97	3.11	3.46	3.44	4.40	4.35	4.11	3.96	3.42	3.37	4.15	4.03	4.26	4.28	1.06	1.13	1.56	1.42	1.07	1.09	0.13	0.18	0.42	0.57	1.11	1.16	0.38	0.50	0.27	0.25
9H	3.50	2.30	2.46	3.26	3.44	2.29	2.30	2.90	3.14	2.63	2.82	3.47	3.29	2.15	2.34	3.13	3.28	1.20	1.04	0.24	0.06	1.21	1.20	0.60	0.36	0.87	0.68	0.03	0.21	1.35	1.16	0.37	0.22
15H	4.15	3.70	3.46	3.88	3.89	3.56	3.56	3.91	3.81	4.14	4.12	3.81	3.84	4.23	4.21	3.85	3.85	0.45	0.69	0.27	0.26	0.59	0.59	0.24	0.34	0.01	0.03	0.34	0.31	0.08	0.06	0.30	0.30
14H	3.38	3.36	3.17	2.85	2.80	3.24	3.21	2.76	2.78	3.48	3.27	2.53	2.57	3.36	3.15	2.50	2.54	0.02	0.21	0.53	0.58	0.14	0.17	0.62	0.60	0.10	0.11	0.85	0.81	0.02	0.23	0.88	0.84
12H	5.54	4.32	4.39	4.83	4.82	4.32	4.32	4.80	4.73	4.76	4.83	4.01	4.03	5.03	5.03	4.19	4.15	1.22	1.15	0.71	0.72	1.22	1.22	0.74	0.81	0.78	0.71	1.53	1.51	0.51	0.51	1.35	1.39
11H	5.11	4.37	4.37	3.55	3.55	3.24	3.24	4.36	4.28	4.21	4.25	3.51	3.53	3.44	3.44	4.42	4.42	0.74	0.74	1.56	1.56	1.87	1.87	0.75	0.83	0.90	0.86	1.60	1.58	1.67	1.67	0.69	0.69
10H	4.50	4.31	4.30	3.86	4.18	4.39	4.36	3.98	4.16	4.49	4.56	4.10	4.05	4.38	4.47	3.94	4.10	0.19	0.20	0.64	0.32	0.11	0.14	0.52	0.34	0.01	0.06	0.40	0.45	0.12	0.03	0.56	0.40
16H	5.97	5.82	5.78	5.94	6.19	5.79	5.81	5.94	6.29	5.89	5.90	6.17	6.01	5.86	5.85	5.84	6.28	0.15	0.19	0.03	0.22	0.18	0.16	0.03	0.32	0.08	0.07	0.20	0.04	0.11	0.12	0.13	0.31
17H	6.57	6.74	7.16	6.97	7.07	7.29	7.14	6.99	6.72	6.76	6.85	7.35	7.33	6.76	7.22	6.97	6.71	0.17	0.59	0.40	0.50	0.72	0.57	0.42	0.15	0.19	0.28	0.78	0.76	0.19	0.65	0.40	0.14
19H	5.96	6.20	6.14	6.18	6.06	6.16	6.15	6.17	6.16	6.16	6.16	6.12	6.14	6.16	6.11	6.16	6.20	0.24	0.18	0.22	0.10	0.20	0.19	0.21	0.20	0.20	0.20	0.16	0.18	0.20	0.15	0.20	0.24
20H	6.15	6.62	6.49	6.47	6.39	6.55	6.50	6.57	6.53	6.61	6.50	6.55	6.61	6.57	6.51	6.53	6.60	0.47	0.34	0.32	0.24	0.40	0.35	0.42	0.38	0.46	0.35	0.40	0.46	0.42	0.36	0.38	0.45
21H	6.11	6.32	6.28	6.33	6.26	6.26	6.25	6.35	6.30	6.29	6.22	6.33	6.33	6.27	6.17	6.35	6.32	0.21	0.17	0.22	0.15	0.15	0.14	0.24	0.19	0.18	0.11	0.22	0.22	0.16	0.06	0.24	0.21
22H	6.06	6.07	6.26	6.00	6.09	6.23	6.26	6.01	6.11	6.11	6.21	6.21	6.29	6.08	6.20	6.00	6.15	0.01	0.20	0.06	0.03	0.17	0.20	0.05	0.05	0.05	0.15	0.15	0.23	0.02	0.14	0.06	0.09
23H	5.29	5.53	5.83	5.58	5.70	6.20	5.74	5.59	5.65	5.50	5.64	6.02	5.78	5.55	5.70	5.63	5.60	0.24	0.54	0.29	0.41	0.91	0.45	0.30	0.36	0.21	0.35	0.73	0.49	0.26	0.41	0.34	0.31
24H	2.79	2.41	2.12	2.54	2.06	2.39	2.35	2.50	2.62	2.41	3.07	2.35	2.88	2.42	2.37	2.47	2.96	0.38	0.67	0.25	0.73	0.40	0.44	0.29	0.17	0.38	0.28	0.44	0.09	0.37	0.42	0.32	0.17
25Ha	2.79	2.11	2.62	2.26	2.58	2.64	2.79	2.19	2.94	2.13	2.75	3.06	3.05	2.17	2.79	2.20	2.87	0.68	0.17	0.53	0.21	0.15	0.00	0.60	0.15	0.66	0.04	0.27	0.26	0.62	0.00	0.59	0.08
25Hb	3.70	3.78	3.89	3.72	3.85	4.25	3.62	3.82	3.23	3.77	3.35	3.80	3.36	3.83	3.60	3.83	3.15	0.08	0.19	0.02	0.15	0.55	0.08	0.12	0.47	0.07	0.35	0.10	0.34	0.13	0.10	0.13	0.55
27H	1.86	1.84	1.75	1.91	1.83	1.79	1.78	1.92	1.84	1.83	1.81	1.83	1.88	1.83	1.81	1.89	1.83	0.02	0.11	0.05	0.03	0.07	0.08	0.06	0.02	0.03	0.05	0.03	0.02	0.03	0.05	0.03	0.03
28H	0.90	0.90	0.96	0.90	0.93	1.22	0.94	0.90	0.90	0.90	0.91	1.11	0.90	0.93	0.95	0.91	0.90	0.00	0.06	0.00	0.03	0.32	0.04	0.00	0.00	0.00	0.01	0.21	0.00	0.03	0.05	0.01	0.00
2Ha	3.78	3.41	3.17	3.34	3.14	3.16	3.23	3.35	3.32	3.38	3.52	3.33	3.49	3.36	3.23	3.32	3.42	0.37	0.61	0.44	0.64	0.62	0.55	0.43	0.46	0.40	0.26	0.45	0.29	0.42	0.55	0.46	0.36
2Hb	3.65	3.20	3.15	3.22	3.14	3.14	3.12	3.19	3.14	3.22	2.97	3.07	3.11	3.18	3.13	3.24	3.12	0.45	0.50	0.43	0.51	0.51	0.53	0.46	0.51	0.43	0.68	0.58	0.54	0.47	0.52	0.41	0.53
4H	6.74	6.74	6.32	6.51	6.30	6.37	6.28	6.49	6.23	6.75	6.17	6.21	6.34	6.73	6.30	6.50	6.21	0.00	0.42	0.23	0.44	0.37	0.46	0.25	0.51	0.01	0.57	0.53	0.40	0.01	0.44	0.24	0.53
5H	7.54	7.92	8.05	7.82	7.97	8.01	7.93	7.86	7.86	7.93	7.62	8.14	7.76	7.90	7.94	7.91	7.82	0.38	0.51	0.28	0.43	0.47	0.39	0.32	0.32	0.39	0.08	0.60	0.22	0.36	0.40	0.37	0.28
7H	6.06	6.90	6.66	6.33	6.54	6.66	6.61	6.45	6.48	6.89	6.61	7.13	6.84	6.78	6.58	6.53	6.87	0.84	0.60	0.27	0.48	0.60	0.55	0.39	0.42	0.83	0.55	1.07	0.78	0.72	0.52	0.47	0.81
26H	1.82	2.03	1.57	1.86	1.64	1.60	1.56	1.95	1.67	1.97	1.60	1.62	1.71	1.93	1.55	1.95	1.67	0.21	0.25	0.04	0.18	0.22	0.26	0.13	0.15	0.15	0.22	0.20	0.11	0.11	0.27	0.13	0.15
																MAE for ^{13}C		2.3	3.4	2.1	3.0	3.5	3.6	2.1	2.1	2.4	3.1	2.8	2.3	2.5	3.4	2.5	2.2
																MAE for ^{1}H		0.39	0.46	0.38	0.42	0.53	0.47	0.33	0.33	0.31	0.31	0.52	0.46	0.35	0.37	0.37	0.37

Table S3. Putative biosynthetic gene cluster of **1**.

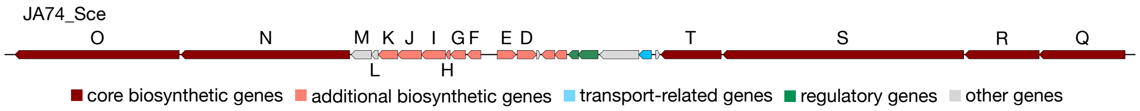

Gene name	Protein length [aa]	Closest homolog[5]	Accession number[5]	Identity [%]	Predicted function[5]
JA74_sceD	411	SceD	ANH11399	80.5	Cytochrome P450 monooxygenase
JA74_sceE	410	SceE	ANH11400	80.8	Cytochrome P450 monooxygenase
JA74_sceF	298	SceF	ANH11401	77.2	Peptidase
JA74_sceG	311	SceG	ANH11402	70.1	Acyl carrier protein aminoacyltransferase
JA74_sceH	83	SceH	ANH11403	60.2	Acyl carrier protein
JA74_sceI	520	SceI	ANH11404	72.5	ATP-dependent aminoacyl-ACP
JA74_sceJ	508	SceJ	ANH11405	71.7	ATP-dependent aminoacyl-ACP
JA74_sceK	412	SceK	ANH11406	74.5	Decarboxylase
JA74_sceL	150	SceL	ANH11407	65.3	Glutamate mutase S subunit
JA74_sceM	429	SceM	ANH11408	72.3	Glutamate mutase E subunit
JA74_sceN	3594	SceN	ANH11409	69.8	Polyketide synthase
JA74_sceO	3503	SceO	ANH11410	74.5	Polyketide synthase
JA74_sceQ	1829	SceQ	ANH11412	70.0	Polyketide synthase
JA74_sceR	1582	SceR	ANH11413	75.1	Polyketide synthase
JA74_sceS	5133	SceS	ANH11414	73.0	Polyketide synthase
JA74_sceT	1284	SceT	ANH11415	72.9	Polyketide synthase

Figure S1. ^{1}H NMR spectrum of streptolactam D (**1**) (500 MHz, Pyridine-d_5).

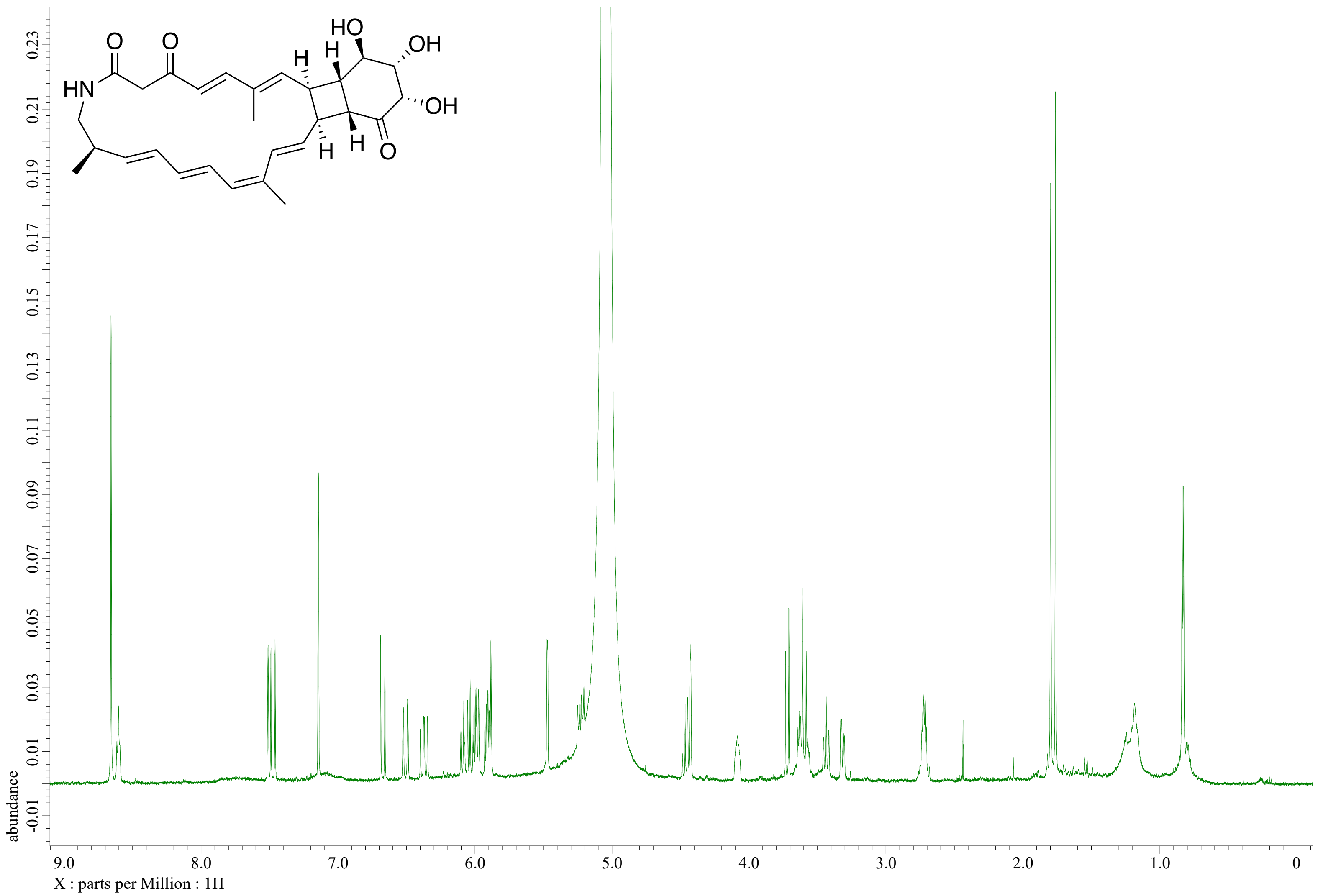

Figure S2. ^{13}C NMR spectrum of **1** (125 MHz, Pyridine-d_5).

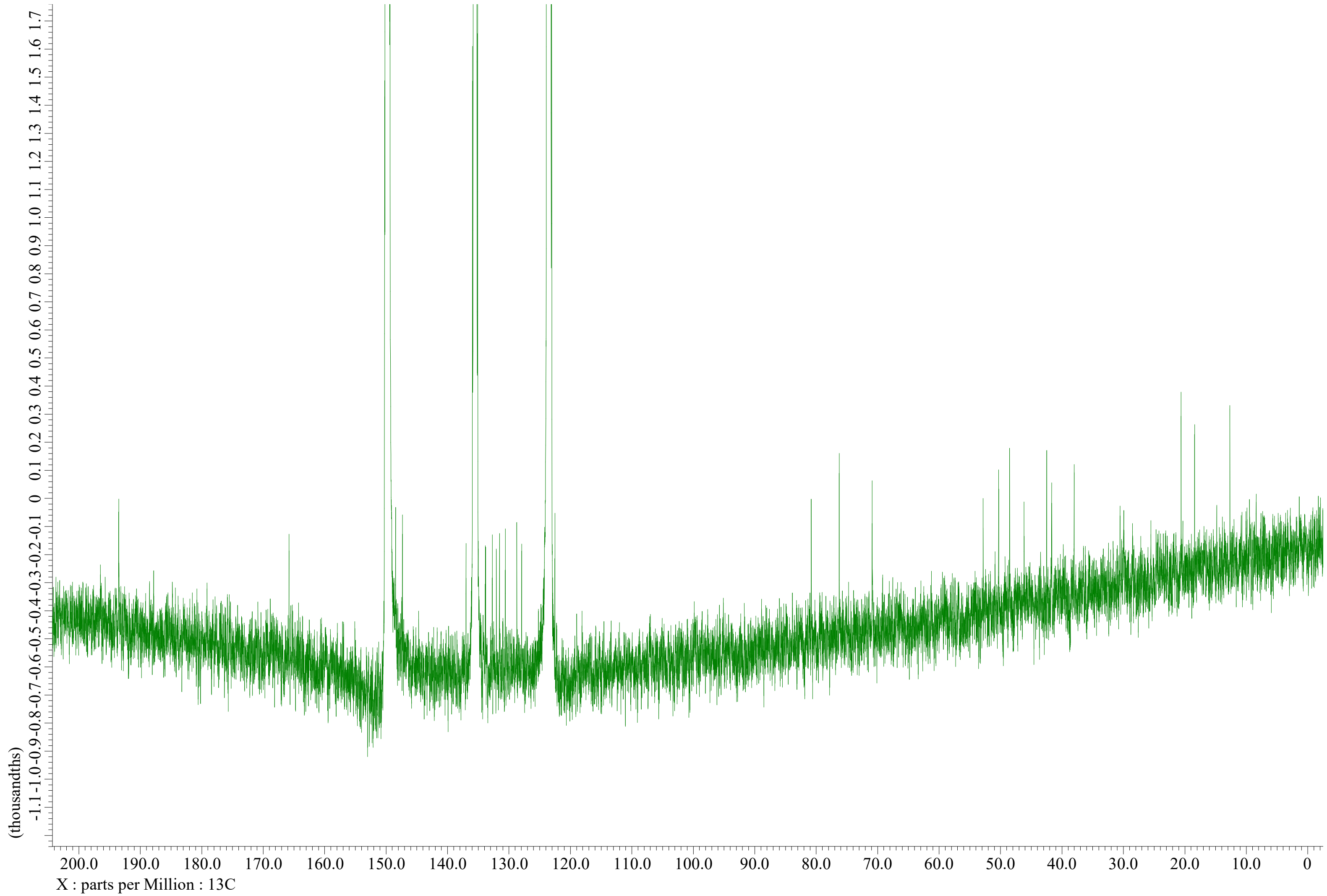

Figure S3. ^{1}H-^{1}H COSY spectrum of **1** (500 MHz, Pyridine-d_5).

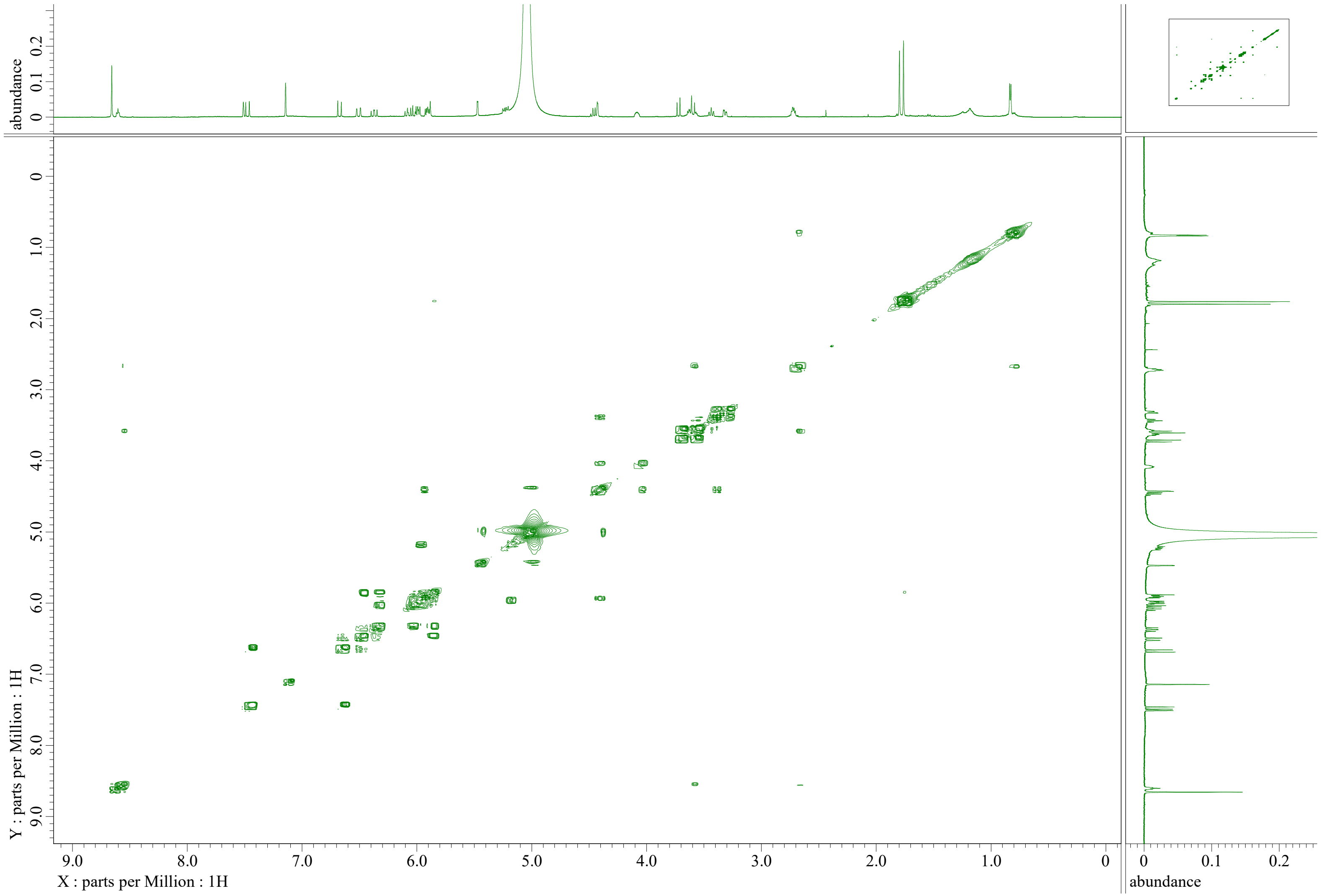

Figure S4. HMQC spectrum of **1** (400 MHz, Pyridine-d_5).

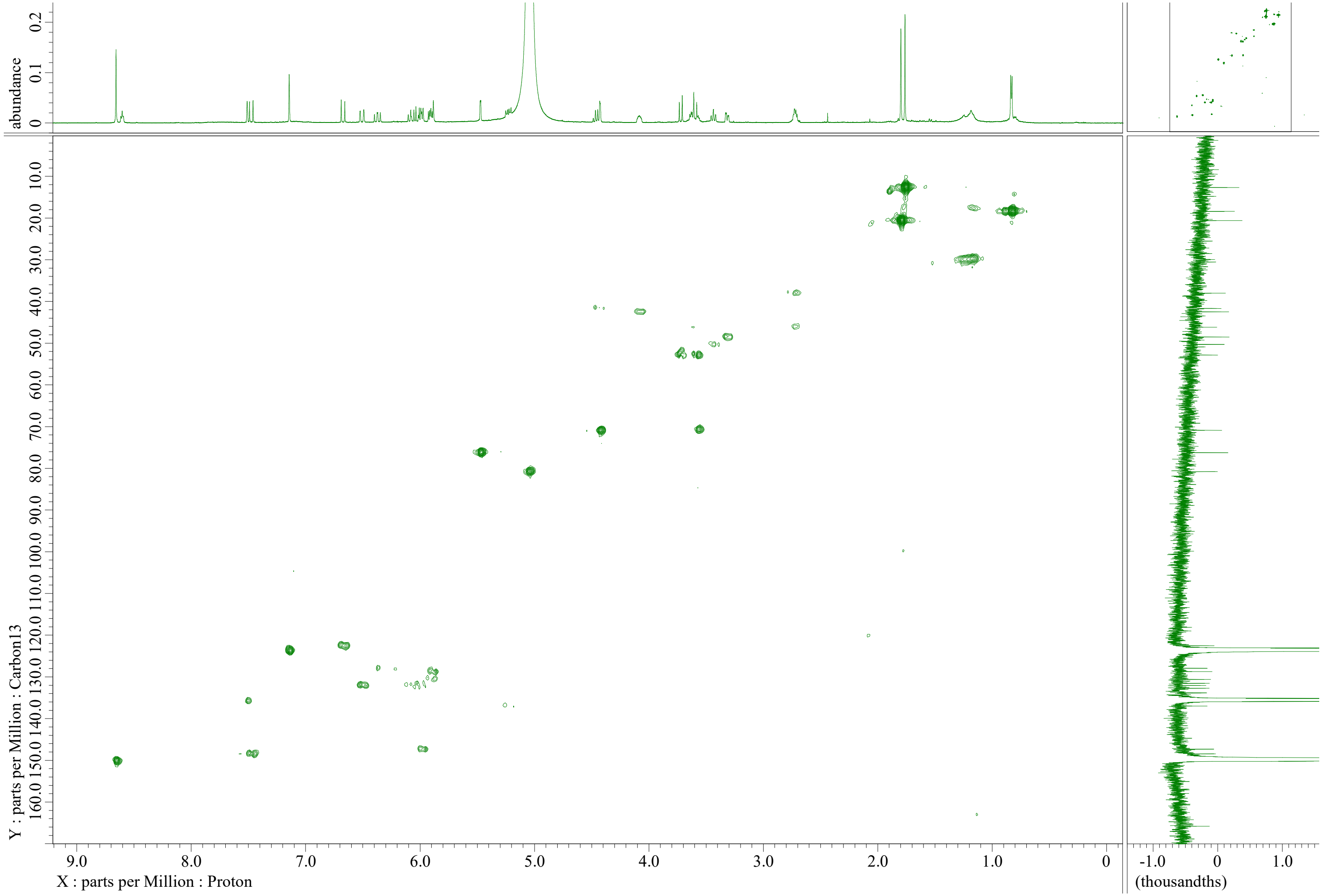

Figure S5. HMBC spectrum of **1** (500 MHz, Pyridine-d_5).

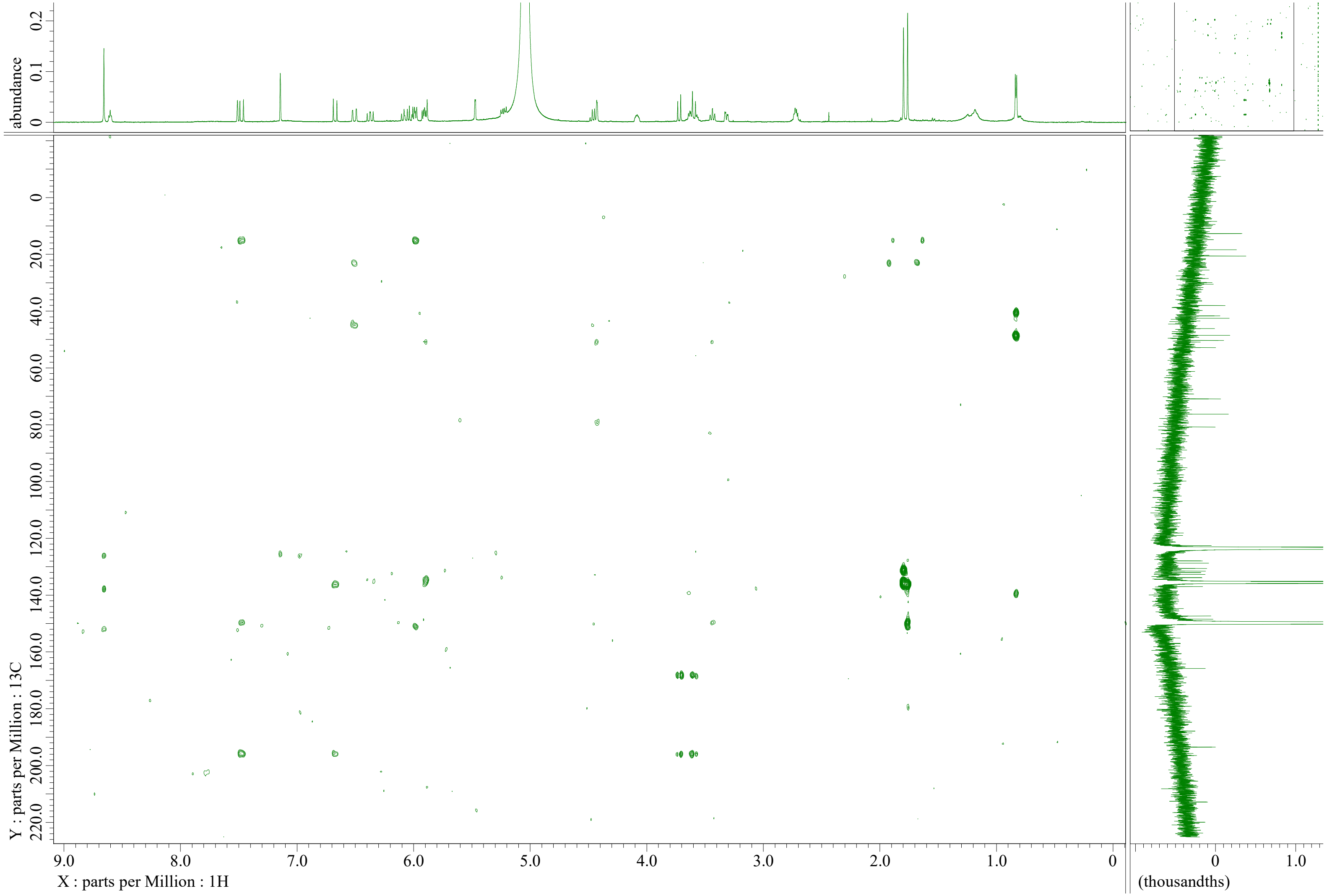

Figure S6. NOESY spectrum of **1** (500 MHz, Pyridine-d_5).

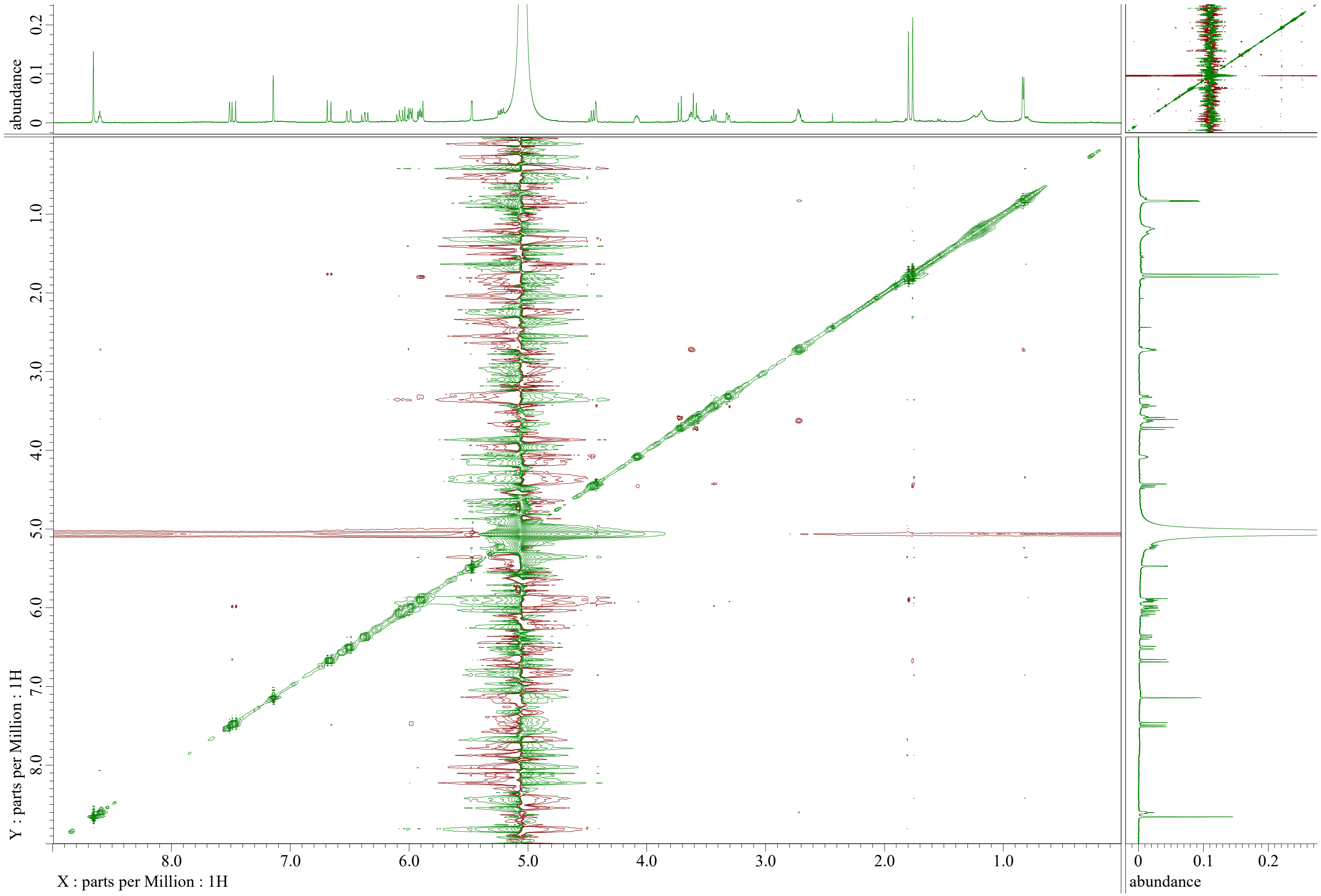

(a)

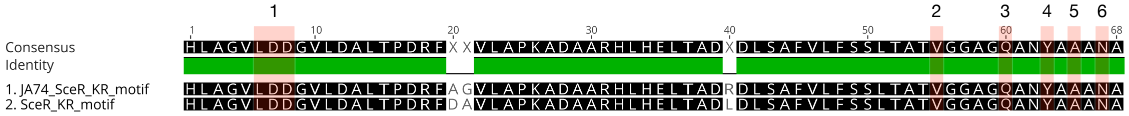

(b)

Substrate	KR type	Product	Fingerprint
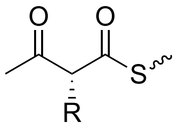	A1	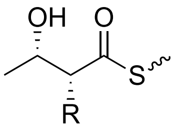	1: no LDD, 2: W, 3: no H
	A2	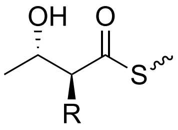	1: no LDD, 2: W, 3: H
	B1	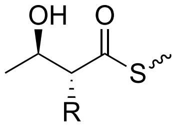	1: LDD, 5: no P
	B2	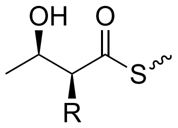	1: LDD, 5: P
	C1	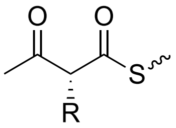	4: no Y
	C2	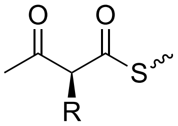	6: < N*<br>*, or obscured dinucleotide binding motif

Figure S7. Sequence analysis of the KR domain. (a) Regulation of stereochemistry by types of KR domain in PKS. (b) Each of the KR domain types. Table (b) was created based on reference number [6].

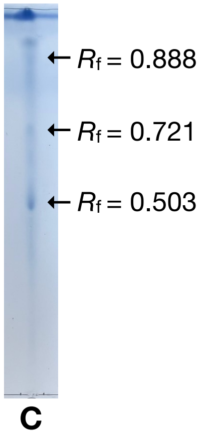

Figure S8. TLC analysis of the cell membrane fraction.

References

[1] Schrödinger Release 2021–2: MacroModel; Schrödinger, LLC: New York, NY, 2021.

[2] M. J. Frisch, G. W. Trucks, H. B. Schlegel, G. E. Scuseria, M. A. Robb, J. R. Cheeseman, G. Scalmani, V. Barone, G. A. Petersson, H. Nakatsuji, X. Li, M. Caricato, A. V. Marenich, J. Bloino, B. G. Janesko, R. Gomperts, B. Mennucci, H. P. Hratchian, J. V. Ortiz, A. F. Izmaylov, J. L. Sonnenberg, D. Williams-Young, F. Ding, F. Lipparini, F. Egidi, J. Goings, B. Peng, A. Petrone, T. Henderson, D. Ranasinghe, V. G. Zakrzewski, J. Gao, N. Rega, G. Zheng, W. Liang, M. Hada, M. Ehara, K. Toyota, R. Fukuda, J. Hasegawa, M. Ishida, T. Nakajima, Y. Honda, O. Kitao, H. Nakai, T. Vreven, K. Throssell, J. A. Jr. Montgomery, J. E. Peralta, F. Ogliaro, M. J. Bearpark, J. J. Heyd, E. N. Brothers, K. N. Kudin, V. N. Staroverov, T. A. Keith, R. Kobayashi, J. Normand, K. Raghavachari, A. P. Rendell, J. C. Burant, S. S. Iyengar, J. Tomasi, M. Cossi, J. M. Millam, M. Klene, C. Adamo, R. Cammi, J. W. Ochterski, R. L. Martin, K. Morokuma, O. Farkas, J. B. Foresman, D. J. Fox, Gaussian 16, Revision C.01; Gaussian, Inc.: Wallingford CT, 2019.

[3] K. Fukaya, ACCeL. https://accel.kfchem.dev/

[4] C. Bannwarth, S. Ehlert, S. Grimme., *J. Chem. Theory Comput.* **2019***, 15,* 1652−1671.

[5] Z. J. Low, L. M. Pang, Y. Ding, Q. W. Cheang, K. L. M. Hoang, H. T. Tran, J. Li, X. W. Liu, Y. Kanagasundaram, L. Yang, Z. X. Liang, *Sci. Rep.* **2018**, *8*, 1594.

[6] A. T. Keatinge-Clay, *Chem. Biol.* **2007**, *14*, 898–908.
